# Bio-Derived Hierarchical Multicore–Shell Fe_2_N-Nanoparticle-Impregnated N-Doped Carbon Nanofiber Bundles: A Host Material for Lithium-/Potassium-Ion Storage

**DOI:** 10.1007/s40820-019-0290-0

**Published:** 2019-07-15

**Authors:** Hongjun Jiang, Ling Huang, Yunhong Wei, Boya Wang, Hao Wu, Yun Zhang, Huakun Liu, Shixue Dou

**Affiliations:** 1 Department of Advanced Energy Materials, College of Materials Science and Engineering, Sichuan University, Chengdu, 610064 People’s Republic of China; 2 Institute for Superconducting and Electronic Materials, Australian Institute of Innovative Materials, University of Wollongong, Wollongong, NSW 2500 Australia

**Keywords:** Anode material, Iron nitride, Lithium-ion battery, Potassium-ion battery, Multicore–shell structure

## Abstract

Despite the significant progress in the fabrication of advanced electrode materials, complex control strategies and tedious processing are often involved for most targeted materials to tailor their compositions, morphologies, and chemistries. Inspired by the unique geometric structures of natural biomacromolecules together with their high affinities for metal species, we propose the use of skin collagen fibers for the template crafting of a novel multicore–shell Fe_2_N–carbon framework anode configuration, composed of hierarchical N-doped carbon nanofiber bundles firmly embedded with Fe_2_N nanoparticles (Fe_2_N@N-CFBs). In the resultant heterostructure, the Fe_2_N nanoparticles firmly confined inside the carbon shells are spatially isolated but electronically well connected by the long-range carbon nanofiber framework. This not only provides direct and continuous conductive pathways to facilitate electron/ion transport, but also helps cushion the volume expansion of the encapsulated Fe_2_N to preserve the electrode microstructure. Considering its unique structural characteristics, Fe_2_N@N-CFBs as an advanced anode material exhibits remarkable electrochemical performances for lithium- and potassium-ion batteries. Moreover, this bio-derived structural strategy can pave the way for novel low-cost and high-efficiency syntheses of metal-nitride/carbon nanofiber heterostructures for potential applications in energy-related fields and beyond.

**Electronic supplementary material:**

The online version of this article (10.1007/s40820-019-0290-0) contains supplementary material, which is available to authorized users.

## Highlights

- A viable bio-derived material engineering strategy is developed based on the use of skin collagen fibers for the crafting of metal-nitride–carbon nanofiber composites.
- N-doped carbon nanofiber bundles embedded with Fe_2_N nanoparticles are fabricated using a structural replication process.
- The composite with a hierarchically ordered, compact, and multicore–shell heterostructure exhibits increased lithium- and potassium-ion storage performances.

**Electronic supplementary material:**

The online version of this article (10.1007/s40820-019-0290-0) contains supplementary material, which is available to authorized users.

## Graphical abstract

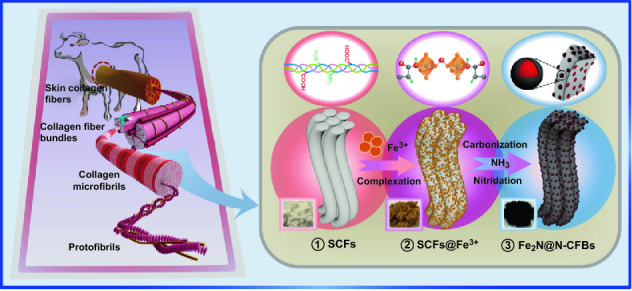

## Introduction

Considering their high energy densities and long lifetimes, lithium-ion batteries (LIBs) have been extensively investigated and widely used as power sources for portable electronics and electric vehicles [1, 2]. However, the scarcity and uneven distribution of lithium resources in the earth restrict their applications. In this regard, potential alternatives have attracted considerable interest. Among them, potassium-ion batteries (PIBs) attract increasing attention, owing to the abundant reserves of potassium on the earth and its standard hydrogen potential (− 2.93 V vs. *E*º) close to that of lithium (− 3.04 V vs. *E*º) [3, 4]. Generally, the main challenge for the PIB anode is the large potassium-ion radius (1.38 Å) [5], leading to sluggish kinetics during electrochemical processes. Although extensive studies have been carried out and significant progress has been made in the development of energy storage materials, the development of advanced anode materials for LIBs and PIBs for large-scale energy storage requires further studies. Transition-metal nitrides (TMNs) have been recently reported as promising conversion anode materials for both LIBs and PIBs, because of their unique physicochemical properties [6] and high theoretical specific capacities [7–10]. Among the TMNs, the earth-abundant and inexpensive iron nitride (Fe_2_N) has attracted considerable attention. As a conversion-type energy storage material, Fe_2_N can reversibly react with Li ions by the formation of metal irons (Fe_2_N + 3Li^+^ + 3e^−^ ↔ 2Fe + Li_3_N) [11, 12]. Owing to its high theoretical density of 7.14 g cm^−3^, it can transfer 2–3 electrons per formula unit, leading to a theoretical specific capacity as high as 900 mAh g^−1^ and high volumetric energy density of the battery [13–16]. Moreover, Fe_2_N exhibits pseudocapacitance and ultrafast charge transfer from surface/subsurface regions because of its stable phase change. Furthermore, Fe_2_N and other metal nitrides exhibit high conductivities and good ionic diffusions owing to the vacancies within their crystal structures. Therefore, it is expected that Fe_2_N as an anode material can be an ideal platform to realize high energy storage capacity and power density. Nonetheless, the practical application of Fe_2_N is still hindered by the common problems of high polarization and large volume changes during lithiation/potassiation processes, inducing a severe electrode pulverization and fast capacity fading as well as short life span during long-term cycling [17, 18].

Nanostructure engineering has paved the way for the development of high-performance Fe_2_N anode materials. Compared to bulk materials, zero-dimensional Fe_2_N nanoparticles (NPs) have more active sites and shorter ionic diffusion paths, while the void space between the particles can help cushion the volumetric changes of the Fe_2_N NPs to some degree [19, 20]. Nevertheless, the simple change in geometric shape and tuning of the particle size are still unsatisfactory for the improvement in cycling lifetime of Fe_2_N, as particle agglomeration is unavoidable and the large volume expansion during the cycling may eventually lead to electrode pulverization. Hierarchically nanostructured composites consisting of nanoscale Fe_2_N and conductive carbon materials could overcome the above drawback to a large extent [12, 21]. Carbon materials such as various forms of amorphous carbon, carbon nanotubes, graphene sheets, and carbon fibers can increase the conductivity, prevent the pulverization of active materials, and reduce the undesirable side reactions between the electrode and electrolyte [17, 22–25]. Nonetheless, this strategy usually involves time-consuming preparation and tedious structure control processes for the composite electrode materials. Therefore, an alternative low-cost and universal approach to the synthesis of uniform Fe_2_N NPs encapsulated in carbon frameworks with desirable compositions and morphologies is desirable.

The inspiration by biological materials with complex, optimized, and hierarchical microstructures has been one of the most promising subjects in artificial material engineering. Generally, the bio-inspired material synthesis strategy is based on the self-assembly of specific guest species to obtain inorganic analogues of the biological materials [26–29]. Natural biological materials used as templates for the material synthesis are low cost, abundant, commercially viable, and environmentally benign [30]. On the other hand, biological materials are morphologically complex but composed of uniform organic/inorganic structural subunits, whose imitation on a small scale could pave the way for the fabrication of novel nanostructured materials. Many biological materials such as biomacromolecules self-assemble into gels or fibers, which can be used to direct the growth of inorganic nanomaterials [31, 32]. In most of these cases, biomacromolecules can provide a matrix, which “traps” metal ions in an aqueous phase. The matrix can be used as a precursor for conversion into nanostructured metal oxides, carbides, or sulfides as well as various carbon-composite heterostructures. For example, carrageenan, extracted from red algae, can strongly bind to multiple metal ions (e.g., Ni^2+^, Co^2+^, Cu^2+^, Fe^3+^) forming carrageenan–metal hydrogels. The organized double-helix structures in the hydrogels could mediate the growth and formation of ultrasmall metal sulfide NPs (e.g., Co_9_S_8_, Ni_3_S_4_, CuS, FeS) into hierarchically porous carbon aerogels by pyrolysis [32]. Therefore, the bio-inspired synthesis routes are unique and effective. However, the fabrication of novel TMN/carbon composites has rarely been reported because it often involves the utilization of toxic and expensive organic ligands as well as time-consuming synthesis routes. Therefore, further development of effective TMN/carbon composites is necessary to address the critical issues related to energy storage.

**Fig. 1:**
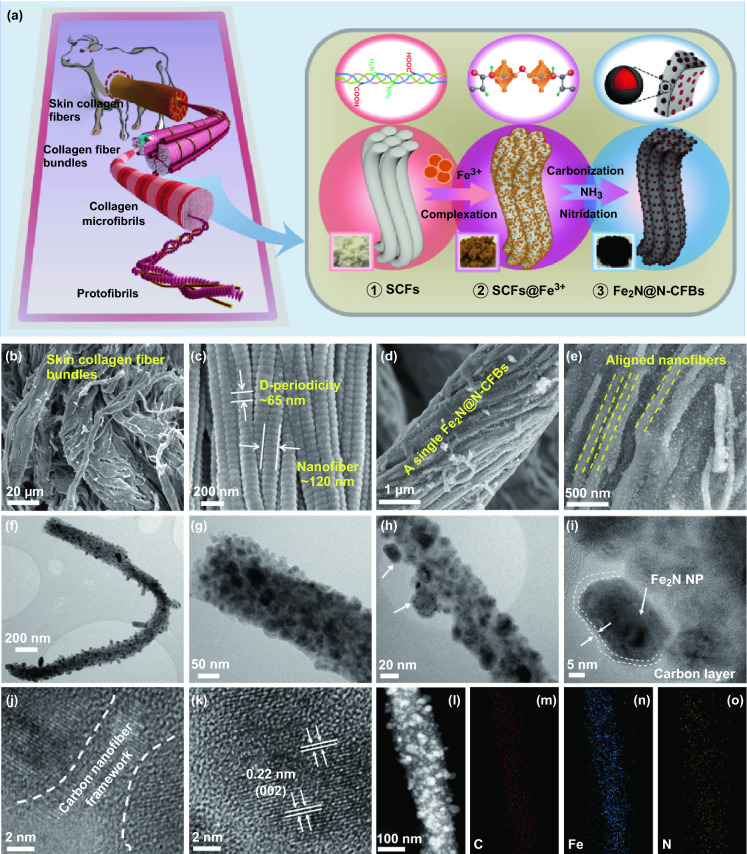
**a** Schematic of the structure of the native SCFs and synthesis strategy for Fe_2_N@N-CFBs. FESEM images of **b**, **c** natural SCFs and **d**, **e** Fe_2_N@N-CFBs. **f**–**i** TEM images of Fe_2_N@N-CFBs at different magnifications. **j**, **k** HRTEM images and **l**–**o** HAADF-STEM image and corresponding element maps of Fe_2_N@N-CFBs

Skin collagen fibers (SCFs), one of the most abundant and renewable biomasses in nature, originate mainly from the skins of domestic animals, which have traditionally been used as raw materials in the leather manufacturing industry [33]. In terms of their geometric structures, the SCFs exhibit a hierarchically interwoven fibrous network morphology. As illustrated in Fig. 1a, the protofibrils are formed from rod-like collagen molecules composed of three polypeptide chains with a triple-helical structure. They are packed together longitudinally in a quarter-staggered alignment with a “gap” region (corresponding to a length of 67 nm) and further organized into larger microfibrils (typical nanofibers with diameters of 50–200 nm). Subsequently, these nanofibrous microfibrils self-assemble into collagen fiber bundles and further into larger SCFs [34]. In their chemical structure, abundant reactive functional groups including –COOH, –OH, and –NH_2_ exist in the side chains of collagen molecules, which provide the SCFs with high affinities toward multivalent metal ions, such as Ni^2+^, Co^2+^, Fe^3+^, Cr^3+^, Al^3+^, and Ti^4+^ [35]. This property suggests that the SCFs can react directly with transition-metal cations in aqueous solutions without the need for further functionalization. Therefore, the unique structural advantages of the SCFs together with their high affinities to inorganic building blocks make them suitable as an ideal template for the development of hierarchically structured composites for conversion-type reaction anode materials of LIBs and PIBs.

To this end, we report a viable material synthesis strategy for the engineering of a novel one-dimensional (1D) multicore–shell Fe_2_N–carbon framework heterostructure for superior LIB and PIB anodes, which is composed of hierarchical N-doped carbon nanofiber bundles firmly embedded with Fe_2_N NPs (denoted as Fe_2_N@N-CFBs). In our design, natural SCFs extracted from cowhide are used not only as the matrix, which can directly react with Fe^3+^ ions in an aqueous solution, but also as the biotemplate for structural replication and conversion to Fe_2_N@N-CFBs through a one-step NH_3_-driven carbonization/nitridation approach, by which the Fe_2_N NPs can be formed and uniformly encapsulated into the SCF-derived carbon nanofiber shells with unique multicore–shell heterostructures. This unique architecture design is expected to provide multiple structural advantages to simultaneously address the drawbacks associated with Fe_2_N during the electrochemical processes. (1) The all-around encapsulation architecture enables a large contact interface between the N-doped carbon nanofiber framework and Fe_2_N NPs along with continuous electron/ion transport pathways in both radial and axial directions, promoting the electrode reaction kinetics. (2) The 1D long-range N-doped carbon nanofiber framework can provide the elastic buffering function to cushion the volume expansion/contraction of the embedded Fe_2_N NPs, preserving the integrity of the electrode microstructure. (3) The compact hierarchical structure of the carbon nanofiber bundles is sufficiently robust to ensure the stable ion intercalation/deintercalation of each internal Fe_2_N@carbon nanofiber contained inside N-CFBs, favoring the cycling stability and high volumetric energy density. The as-developed Fe_2_N@N-CFB electrode exhibits high reversible capacities of 660 mAh g^−1^ at 100 mA g^−1^ for LIBs and 242 mAh g^−1^ at 50 mA g^−1^ for PIBs. A remarkable rate performance (202 mAh g^−1^ at 10.0 A g^−1^) and ultralarge cycling lifetime (580 and 420 mAh g^−1^ after 1000 cycles at 1.0 and 2.0 A g^−1^, respectively) in LIBs have also been achieved.

## Experimental Section

### Preparation of Materials

Raw materials: White SCFs were prepared using bovine hides according to the previously reported method [36]. The bovine hides were cleaned, unhaired, limed, split, and delimed according to the procedures of leather processing to remove noncollagen components. The skin was then treated with an aqueous solution of acetic acid (16.0 g L^−1^) three times to remove mineral substances. After the pH of the skin was adjusted to 4.8–5.0 with an acetic acid–sodium acetate buffer solution, the skin was dehydrated by absolute ethyl alcohol, dried in vacuum, ground, and sieved, yielding the SCFs. A chemically pure Fe_2_(SO_4_)_3_ was purchased from the Jinshan Chemical Reagent Factory. Analytical-grade sodium chloride, acetone, and other reagents were purchased from the Chengdu Kelong Chemical Reagent Factory.

Immobilization of Fe^3+^ on SCFs: 15.0 g of collagen fibers and 6.0 g of NaCl were soaked in 400 mL of deionized water at 25 °C. After continuous stirring for 2 h, the pH value of the suspension was adjusted to 1.7−2.0 using a diluted H_2_SO_4_ (1 mol L^−1^). Subsequently, 22.5 g of Fe_2_(SO_4_)_3_ was added and the suspension was kept constant under stirring for 4 h. The pH value was then slowly increased to ≈ 3.8–4.0 using a saturated sodium bicarbonate solution over more than 2 h. The resultant mixture was reacted at 40 °C for 4 h. Finally, the Fe^3+^-immobilized SCFs (SCFs@Fe^3+^) were collected by filtration, followed by washing with deionized water and acetone and drying in air.

Preparation of the Fe_2_N@N-CFB composite: The as-prepared SCFs@Fe^3+^ was then treated in a tube furnace with a programmable control of the temperature. The sample was heat-treated stepwise, first at 100 °C in air for 1 h, and subsequently heated to 500 °C (2 °C min^−1^) in an NH_3_ atmosphere and held at this temperature for 2 h to generate Fe_2_N-nanoparticle-impregnated N-doped carbon nanofiber bundles (Fe_2_N@N-CFBs). For comparison, pure N-doped carbon nanofiber bundles (N-CFBs) were also prepared using HF etching to remove the Fe_2_N NPs from Fe_2_N@N-CFBs. Bare Fe_2_N nanofiber bundles (Fe_2_N-FBs) were also prepared by a simple two-step calcination method, i.e., SCFs@Fe^3+^ was precalcined in air for 4 h to remove the organic framework, and then the resultant products were treated in an NH_3_ atmosphere at 500 °C to yield bare Fe_2_N-FBs.

### Material Characterization

The crystal structure of the product was analyzed by X-ray diffraction (XRD, Bruker) with Cu K_α_ radiation (*λ* = 1.54056 Å). The chemical states and compositions of the samples were characterized by X-ray photoelectron spectroscopy (XPS) using an ESCALAB 250Xi instrument; all binding energy peaks were calibrated by C 1*s* at 284.5 eV. The peak fittings of all high-resolution core spectra were carried out with the XPSPEAK 4.1 software, using a mixed Gaussian–Lorentzian function. The morphologies and microstructures of the products were observed using field-emission scanning electron microscopy (FESEM, Nova NanoSEM 450), coupled with energy-dispersive X-ray (EDX, Oxford Instrument) spectroscopy, and transmission electron microscopy (TEM, JEOL, JEM-2100F, 200 kV, equipped with an EDX spectrometer). A thermogravimetric analysis (TGA) was carried out using a Netzsch TG209F1 instrument in the range of room temperature to 800 °C at a heating rate of 10 °C min^−1^ under air atmosphere. Differential scanning calorimetry was performed using a NETZSCH STA 449C instrument in the range of room temperature to 800 °C (10 °C min^−1^) under Ar atmosphere. Raman spectra were recorded using a JY HR800 Raman spectrophotometer (HORIBA Jobin Yvon, HR800, France) with a 532.17-nm laser radiation. Mössbauer measurements were performed using a conventional spectrometer (Germany, Wissel MS-500) in the transmission geometry and constant acceleration mode. A ^57^Co(Rh) source with an activity of 25 mCi was used. The velocity calibration was carried out with a room-temperature α-Fe absorber. The spectra were fitted by the Recoil software package using a Lorentzian site analysis.

### Electrochemical Measurements

Electrochemical measurements were carried out using lithium-ion half-cells. The working electrodes were prepared by mixing the active material, Super P, and polyvinylidene fluoride in a mass ratio of 7:2:1 in an N-methyl-2-pyrrolidone solvent, and then the mixed slurry was spread onto a copper foil. The as-prepared electrodes were dried in vacuum at 60 °C for 12 h. The mass loading of active material on each electrode was 1.0 −1.2 mg cm^−2^. Two-electrode CR2032 coin cells were assembled in an argon-filled glove box to evaluate the electrochemical performances of the samples. In the LIBs, the counter electrode was a disk of lithium foil, while a porous polypropylene film (Celgard 2400) served as the separator. The electrolyte was 1 M of LiPF_6_ dissolved in a mixture of ethylene carbonate (EC), ethyl methyl carbonate, and dimethyl carbonate (DEC) in a volume ratio of 1:1:1. In the case of the PIBs, CR 2016 coin-type cells were assembled. The electrolyte consisted of a solution of 0.8 M of KPF_6_ in EC/DEC (volume ratio of 1:1). The counter and reference electrodes were potassium metal, and the separator was a glass fiber. All coin cells were aged at room temperature for 12 h before the test to ensure that the electrode was completely soaked in the electrolyte. Galvanostatic charge/discharge tests were carried out using a Neware battery test system (Neware BTS, Neware, Shenzhen, China) in a voltage window of 0.01−3.0 V. Cyclic voltammetry (CV) measurements were performed at a scan rate of 0.1 mV s^−1^ in the range of 0.01−3.0 V using a PARSTAT multichannel electrochemical working station (Princeton Applied Research, USA). Electrochemical impedance spectroscopy (EIS) was carried out using a PARATAT electrochemical workstation with an alternating-current (AC) voltage amplitude of 5 mV in the frequency range of 100 kHz to 0.1 Hz.

Calculation of volumetric capacity: The mass density of the electrode was calculated by *ρ* = *M* (mg)/*V* (cm^3^), where *M* and *V* are the mass and volume of the electrode, respectively. The thickness of the electrode was estimated by FESEM. The volumetric capacity (*C*_V_) was calculated by *C*_V_ = *C*_g_ × *ρ*.

## Results and Discussion

### Fabrication and Structural Characterization

The overall synthesis strategy for the hierarchically ordered multicore–shell Fe_2_N@N-CFBs is illustrated in Fig. 1a. First, the Fe^3+^ precursors can be directly adsorbed and immobilized on the SCFs in the aqueous solution, in which the Fe^3+^ were primarily reacted and coordinated with the –COOH groups in the collagen triple helix through the formation of coordination complexes [37], as indicated by the color change of the SCFs from white to yellow. After the thermal treatment of the resultant SCFs@Fe^3+^ intermediates in the NH_3_ atmosphere at 500 °C, the organic SCF framework can be completely decomposed, leaving the N-doped carbon nanofiber bundles, while the coordinated Fe^3+^ ions were able to react with the active nitrogen released from NH_3_ through in situ conversion into Fe_2_N NPs embedded in the SCF-derived carbon nanofiber during the high-temperature nitridation. A unique multicore–shell Fe_2_N@N-CFB sample was finally obtained. For comparison, two control samples were prepared. One of them consisted of pure N-doped carbon nanofiber bundles (denoted as N-CFBs) obtained by etching out all Fe_2_N NPs from the Fe_2_N@N-CFBs, while the other sample was bare Fe_2_N nanofiber bundles (denoted as Fe_2_N-FBs) prepared by thermal removal of the SCF organic framework. The corresponding preparation is presented in detail in Experimental Section.

The morphologies and microstructures of the samples were analyzed using FESEM. As shown in Fig. 1b, c, the basic building blocks of the native SCFs are the collagen nanofibers with a distinctive *D*-period structure (length: ≈ 65 nm) and average diameter of 50–200 nm (Fig. 1c), which self-assemble into a collagen microfibril and even into a larger collagen fiber bundle (5–10 μm, Fig. 1b), confirming the intrinsic hierarchically fibrous structure of the SCFs from the nanoscale to the microscale. Similar to the native SCFs (Fig. 1b, c), it should be noted that the SCFs@Fe^3+^ intermediates consisting of closely packed nanofibers also exhibit a distinctly fibrous morphology (Fig. S1), suggesting that the Fe^3+^-complexing process did not destroy the bio-inherent hierarchical structure of the SCFs. The FESEM image of Fe_2_N@N-CFBs shows that a single fiber bundle with an average diameter of 3–5 µm is composed of densely packed nanofibers (Fig. 1d). The nanofibers with smaller diameters of 50–100 nm are aligned together in a definite and ordered arrangement. Fe_2_N@N-CFBs still maintains all basic building blocks of the nanofiber morphology of the SCF template. Moreover, the magnified FESEM image in Fig. 1e shows a large number of small Fe_2_N NPs on the surface of each nanofiber. To further demonstrate the existence of Fe_2_N NPs, Fe_2_N@N-CFBs was further subjected to etching by hydrofluoric acid. As shown in Fig. S2, after the treatment by acidic etching, the resultant N-CFBs still consisted of numerous interwoven nanofibers, each of which had a relatively smooth surface, along with a large number of visible pores (marked by yellow circles in Fig. S2b). This implies that the Fe_2_N NPs can be removed from Fe_2_N@N-CFBs. They can serve as in situ pore-forming agents to prepare porous carbon nanofiber bundles. It is worth mentioning that the native SCFs without Fe^3+^-coordination cannot retain their natural fibrous structure if they are carbonized directly under the NH_3_ atmosphere at 500 °C, as shown in Fig. S3. According to the principles of the traditional mineral tanning chemistry, the tanning interaction of animal hides and skins with metal salts could significantly increase the degree of cross-linking between the collagen macromolecules, thereby improving the thermal shrinkage temperature of collagen (Fig. S4) and leading to their transformation into leather. Therefore, our results also confirm that the precoordination to Fe^3+^ is indispensable to maintain and stabilize the fibrous structure of the native SCFs to ensure their conversion to ordered CFBs during the high-temperature carbonization process [38].

To further demonstrate the applicability of the SCFs as a template, the Fe^3+^-immobilized SCFs were directly calcined in air to convert them into Fe_2_O_3_ intermediates (Fig. S5) prior to the nitridation treatment. Owing to the template replication action of the SCFs, the resultant Fe_2_N-FBs composed of numerous Fe_2_N particles also exhibited a unique fibrous morphology similar to that of the pristine SCFs, as shown in Fig. S6. In contrast to Fe_2_N@N-CFBs, it should be noted that the bare Fe_2_N-FBs had a denser and more compact structure and thicker fiber bundles. This indicates that despite the effective replication of the fibrous structure, the absence of the SCF-derived carbon framework led to the conspicuous agglomeration of Fe_2_N particles in the bare Fe_2_N-FBs. Figure S7 shows SEM–EDX spectroscopy maps, which verify the uniform distributions of Fe, N, C, and small amount of O element in Fe_2_N@N-CFBs.

The microstructure of Fe_2_N@N-CFBs was further analyzed by TEM. Typical TEM images of an individual nanofiber are presented in Fig. 1f, g. The diameter of the individual nanofiber is approximately 100 nm. Numerous well-crystallized Fe_2_N NPs (black regions) with an average size of ≈ 10–20 nm are uniformly dispersed over and confined within the nanofiber, suggesting the unique multicore–shell nanostructure of Fe_2_N@N-CFBs. Some Fe_2_N NPs are dispersed over the outer surface of the nanofiber, as shown in Fig. 1h (marked by white arrows). To analyze the surroundings of these Fe_2_N NPs, high-resolution TEM (HRTEM) observations were performed. Figure 1i shows a uniform and thin carbon layer with a thickness of ≈ 5 nm covering the Fe_2_N NP clinging to the nanofiber matrix, which provides a charge transfer interphase enabling a good contact between the carbon nanofiber and Fe_2_N NPs. Moreover, as shown in Fig. 1j, the Fe_2_N NPs confined in the thin carbon shells are geometrically isolated from each other by the SCF-derived carbon nanofiber framework, which can also act as an ideal elastic matrix to accommodate the volume expansion of the enclosed Fe_2_N NPs. In addition, the HRTEM image (Fig. 1k) shows clear lattice fringes with an interplanar spacing of 0.221 nm, well corresponding to the (002) lattice spacing of Fe_2_N. To further demonstrate the uniform dispersion of the Fe_2_N NPs, a dark-field image (Fig. 1l) was acquired using high-angle annular dark-field scanning TEM (HAADF-STEM). The EDX spectroscopy elemental mapping corresponding to the HAADF-STEM image (Fig. 1m–o) shows uniform distributions of the Fe, C, and N elements in Fe_2_N@N-CFBs. Furthermore, Figs. S8 and S9 show TEM and HRTEM images of the two control samples, N-CFBs and Fe_2_N-FBs, respectively. Notably, a remarkably porous texture and oval pore tunnels can be observed in N-CFBs by the apparent contrast (marked with white arrows, Fig. S8c), originating from the in situ removal of the Fe_2_N NPs from the carbon nanofiber by the HF etching. This is consistent with the FESEM observations (Fig. S2), further verifying that the Fe_2_N NPs are embedded in the carbon nanofiber with a multicore–shell nanostructure. The TEM images (Fig. S9) of the bare Fe_2_N-FBs reveal that a single Fe_2_N fiber is composed mainly of aggregated Fe_2_N crystals exhibiting clear lattice fringes separated by 0.21 nm corresponding to the (011) lattice planes of Fe_2_N.

**Fig. 2:**
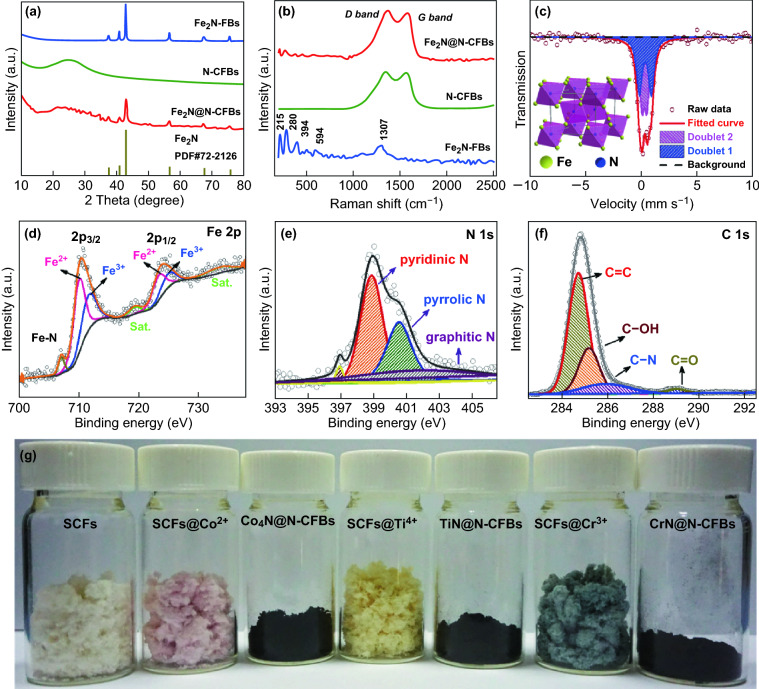
**a** XRD patterns and **b** Raman spectra of Fe_2_N@N-CFBs, N-CFBs, and Fe_2_N-FBs. **c** Mössbauer spectrum of Fe_2_N@N-CFBs at room temperature. The inset shows the crystal structure of the orthorhombic Fe_2_N. **d**–**f** HR XP spectra of the Fe 2p, N 1s, and C 1s peaks of Fe_2_N@N-CFBs. **g** Photographs of SCFs, SCFs@M^*n*+^, and M_*x*_N_*y*_@N-CFBs (M = Co, Ti, Cr)

The crystalline structures of the as-prepared Fe_2_N@N-CFBs and control samples were investigated by powder XRD. As presented in Fig. 2a, three major characteristic peaks at 40.9°, 42.9°, and 56.7° were observed for Fe_2_N-FBs and Fe_2_N@N-CFBs. These peaks can be attributed to the (002), (111), and (112) planes of the ideal Fe_2_N (JCPDS No. 72-2126), respectively, demonstrating the existence of Fe_2_N in Fe_2_N@N-CFBs. Moreover, the diffraction peaks of Fe_2_N@N-CFBs are smaller than those of the bare Fe_2_N-FBs, indicating the smaller sizes of the Fe_2_N NPs in Fe_2_N@N-CFBs. Additionally, compared with the bare Fe_2_N-FBs, the diffraction hump between 20° and 30° in the XRD patterns of Fe_2_N@N-CFBs and pure N-CFBs is attributed to the carbon nanofiber framework. These results indicate the formation of well-crystallized Fe_2_N NPs as well as N-CFBs. Figure 2b presents the Raman spectra of the pure N-CFBs, bare Fe_2_N-FBs, and Fe_2_N@N-CFBs. For the Fe_2_N@N-CFBs composite, the peaks below 1000 cm^−1^ match well with those of the bare Fe_2_N-FBs. For the pure N-CFBs and Fe_2_N@N-CFBs, the two characteristic broad peaks at approximately 1355 and 1577 cm^−1^ are related to the *sp*^3^-type disordered carbon (D band) and *sp*^2^-type ordered graphitic carbon (G band), respectively [39]. According to the calculation, the intensity ratio of the D band to the G band (*I*_D_/*I*_G_) is 1.12, which reveals that the carbon nanofibers have more defects and more disordered structure after the N-doping. To further identify the exact phase of Fe_2_N, we employed Mössbauer spectrometry to study the different types of Fe environments in Fe_2_N@N-CFBs (Fig. 2c). At room temperature, the spin relaxation time of superparamagnetic NPs is on the order of 10^−11^ to 10^−12^ s, considerably smaller than the nuclear sensing time (10^−8^ s), and the sextet collapses into a singlet or doublet [40]. The spectrum of Fe_2_N@N-CFBs exhibits an asymmetric doublet, indicating its paramagnetic nature. The spectral areas of the two doublets are related to the formation probabilities of the two iron sites. The formation of the Fe-III site is associated with additional nitrogen neighbors of iron, which enables an increase in the isomer shift. The formation of the Fe-II site implies a slight deviation from the ideal Fe_2_N stoichiometry, i.e., an orthorhombic ζ-Fe_2_N_1−*z*_ phase (inset in Fig. 2c). Therefore, the pure paramagnetic spectrum of ζ-Fe_2_N_1−*z*_ has to be further fitted with two subspectra for the Fe-III and Fe-II sites, provided that *z* ≠ 0 [41, 42]. For Fe_2_N@N-CFBs, the peak area ratio of the Fe-III site to the Fe-II site is 51.4:48.6, corresponding to *z* of 0.16 and formula of Fe_2_N_0.84_. This shows that Mössbauer spectrometry is an accurate approach to determine the phase compositions of Fe_2_N materials. The weight fraction of Fe_2_N in Fe_2_N@N-CFBs was also estimated by a TGA, as shown in Fig. S10. Considering that the Fe:N ratio is 2:0.84, the Fe_2_N content in Fe_2_N@N-CFBs is estimated to be 41.5%.

The surface element composition and chemical state of Fe_2_N@N-CFBs were further investigated by XPS. The survey scan XP spectrum (Fig. S11) shows the typical Fe, N, C, and O signals, without signals of impurities. The high-resolution XP spectrum of the Fe 2*p* orbitals (Fig. 2d) exhibits broad peaks around 711 and 725 eV corresponding to the Fe 2*p*_3/2_ and Fe 2*p*_1/2_ orbitals, respectively. The peaks at 710.3 and 723.6 eV are related to Fe^2+^, those at 711.9 and 725.5 eV are attributed to Fe^3+^, and those at 719.2 and 734.1 eV are attributed to satellite peaks (Sat.) [20, 21]. These results indicate that the valence state of Fe in Fe_2_N@N-CFBs is a mixture of +2 and +3. The high-resolution N 1*s* XPS peak was further deconvoluted (Fig. 2e), and three types of nitrogen were identified, pyridinic at 398.4 eV, pyrrolic at approximately 400.1 eV, and graphitic at 401.1 eV [43, 44]. Majority (52%) of the nitrogen in Fe_2_N@N-CFBs belongs to the pyridinic type, which could facilitate the Li-ion intercalation into the carbon [45]. The peak around 397.1 eV is associated with the characteristic peak of metal nitride [46]. The high-resolution XP spectrum of C 1*s* (Fig. 2f) is further resolved into four peaks at 284.2, 284.6, 285.4, and 288.5 eV corresponding to C=C, C–OH, C–N, and C=O bonds, respectively [47]. The residual oxygen-containing functional groups originating from the carbonization of the organic SCFs are helpful to increase the surface wettability of the electrode.

**Fig. 3:**
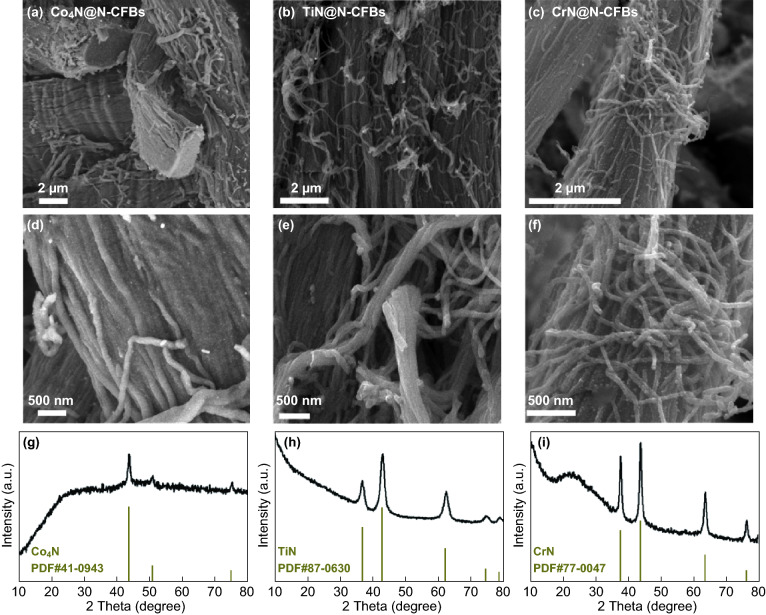
FESEM images of **a**, **d** Co_4_N@N-CFBs, **b**, **e** TiN@N-CFBs, and **c**, **f** CrN@N-CFBs prepared using SCFs as templates and **g**–**i** corresponding XRD patterns

The chemical structure of the collagen macromolecule enables its reactions with various metal salts, such as Co^2+^, Fe^3+^, Cr^3+^, Al^3+^, and Ti^4+^, which are also the fundamental chemical processes for the traditional leather production technology. This inspires us to propose a universal and generalizable synthesis strategy based on the use of the native SCFs as the biotemplate for the engineering of other metal-nitride/carbon nanofiber bundle composites (M_*x*_N_*y*_@N-CFBs, where M is a metal). As shown in Fig. 2g, when the Fe^3+^ precursors were replaced by Co^2+^, Ti^4+^, and Cr^3+^ precursors, the corresponding SCFs@M^*n*+^ intermediates can be obtained. As expected, we also successfully prepared various M_*x*_N_*y*_@N-CFBs products including Co_4_N@N-CFBs (JCPDS No. 41-0943), TiN@N-CFBs (JCPDS No. 87-0630), and CrN@N-CFBs (JCPDS No. 77-0047), which can be verified by the corresponding XRD patterns (Fig. 3g–i). Their typical FESEM images shown in Fig. 3a–f demonstrate the similar morphological characteristics to those of Fe_2_N@N-CFBs. Similar 1D hierarchical nanofiber bundle structures can be well obtained using an identical method, while metal particles are uniformly dispersed on each of the carbon nanofibers. Therefore, the proposed SCF-based synthesis approach is versatile and may lead to more convenient and competitive routes for a generalizable fabrication of various metal-nitride-based hierarchically heterostructured electrode materials toward potential energy-related applications.

### Electrochemical Performances in Lithium-Ion Storage

**Fig. 4:**
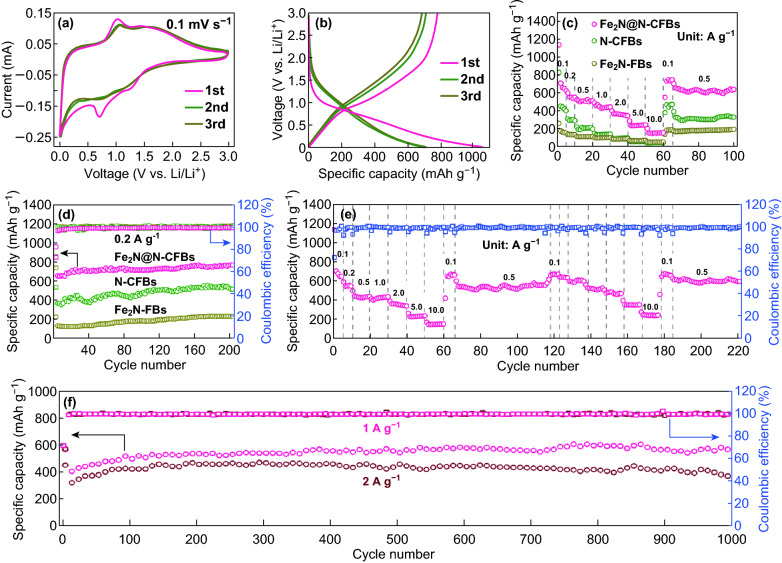
Lithium-ion storage properties of Fe_2_N@N-CFBs. **a** CV curves and **b** discharge–charge voltage profiles of Fe_2_N@N-CFBs for the first three cycles. **c** Cycling performances and **d** rate capacities of Fe_2_N@N-CFBs, N-CFBs, and Fe_2_N-FBs. **e** Repeated rate and cycling tests and **f** long-term cycling performance of Fe_2_N@N-CFBs

The lithium storage behavior of the as-obtained Fe_2_N@N-CFBs was initially investigated by CV at a scan rate of 0.1 mV s^−1^ in the voltage range of 0.01–3.0 V (vs. Li/Li^+^). As shown in Fig. 4a, the small reduction peak at approximately 1.25 V in the first cathodic scan, which disappears in the subsequent cycles, corresponds to the formation of the solid electrolyte interface (SEI) by the irreversible reaction with the electrolyte [12, 21], while the large reduction peak at approximately 0.7 V can be attributed to the lithiation reactions of Fe_2_N (Fe_2_N + 3Li^+^ + 3e^−^ → 2Fe + Li_3_N). During the following anodic scan, a broad oxidation peak is observed around 1.0 V, which could be attributed to the oxidation of Fe to Fe_2_N species (2Fe + Li_3_N → Fe_2_N + 3Li^+^ + 3e^−^). Notably, an obvious difference between the first and following cycles is observed, i.e., the cathodic peak is smaller after the first cycle, which is attributed to the polarization of the electrode in the initial cycle. Nevertheless, both cathodic and anodic peaks in the CV curves overlap with small changes in the subsequent cycles, indicating the excellent reversibility of the electrode. A similar behavior is observed in the CV curves of the bare Fe_2_N-FBs and N-CFBs electrodes (Fig. S12). Figure 4b shows typical galvanostatic discharge/charge curves for the initial three cycles of the Fe_2_N@N-CFBs electrode at a current density of 0.1 A g^−1^; the specific capacities were calculated based on the total mass of Fe_2_N@N-CFBs. In the first cycle, Fe_2_N@N-CFBs delivers high initial discharge and charge capacities of 1062 and 781 mAh g^−1^, respectively, suggesting an initial Coulombic efficiency (ICE) of 73.5%. The irreversible capacity loss can be attributed mainly to the formation of the SEI layer and irreversible insertion of Li^+^ into the defects in the carbon fiber [30]. Subsequently, the electrode delivers discharge capacities of 712 and 698 mAh g^−1^ in the second and third cycles, while the corresponding CE quickly increases to 97.4% and 98.6%, respectively, indicating a relatively stable charge/discharge process with a good electrochemical reversibility. Notably, the ICE of the Fe_2_N@N-CFBs electrode is considerably higher than those of the Fe_2_N-FBs (68.7%) and N-CFBs (46.3%) electrodes (Fig. S13). This demonstrates that the unique 1D hierarchical structure of N-CFBs and thin carbon layer can serve as ideal conductive substrate and confinement of the active Fe_2_N NPs to enable the stable and uniform SEI formation. The increased ICE of the Fe_2_N@N-CFBs electrode may also be partially associated with the proper surface area and pore size distribution compared with those of the other two electrodes, as shown in Fig. S14. In addition, based on the carbon content (58.5%) as well as the specific capacities of the Fe_2_N@N-CFBs composite (698 mAh g^−1^) and pure N-CFBs (452 mAh g^−1^) at the third cycle (Fig. S13b), the reversible Li^+^ storage capacity originating from the Fe_2_N is 434 mAh at 0.1 A g^−1^ with the deduction in capacity contribution of N-CFBs. The corresponding utilization efficiency of active Fe_2_N materials in the Fe_2_N@N-CFBs electrode is then calculated to be 48%, provided that the theoretical specific capacity of Fe_2_N is 900 mAh g^−1^. This value is significantly higher than that obtained for the Fe_2_N-FBs electrode (18%), confirming the crucial role of the N-CFBs substrate acting as the 1D hierarchical dispersant and excellent electric conductor for the active Fe_2_N NPs.

Figure 4c shows the cycling performances of the Fe_2_N@N-CFBs electrode at a current density of 0.2 A g^−1^. Surprisingly, the Fe_2_N@N-CFBs electrode maintains a high reversible capacity of 710 mA h g^−1^ after 200 cycles without decay. In contrast, the bare Fe_2_N-FBs and pure N-CFBs electrodes exhibit relatively low capacities of 220 and 452 mAh g^−1^, respectively. Further EIS tests indicate that the Fe_2_N@N-CFBs electrode not only has the smallest interfacial resistance (*R*_ct_, Fig. S15), but also exhibits the highest Li^+^ diffusion coefficient during 200 cycles (Fig. S16). Furthermore, we observed a gradual decrease in *R*_ct_ for both Fe_2_N@N-CFBs and Fe_2_N-FBs electrodes. In particular, a large decrease from ≈ 90 Ω to only 25 Ω after 200 cycles is observed for the Fe_2_N@N-CFBs electrode. The continuous decrease in charge transfer resistance and capacity increase during the cycling may be attributed to the gradual activation and electrochemical milling processes, where the inner Fe_2_N NPs and N-CFBs are gradually wetted by the electrolyte and thus provide sufficient active sites for the surface electrochemical reaction as well as faster electrode/electrolyte interface kinetics because of the efficient contact with the electrolyte. In addition to its remarkable cycling performance, the rate capabilities of Fe_2_N@N-CFBs were evaluated at various current densities. As shown in Fig. 4d, Fe_2_N@N-CFBs delivered high specific capacities of 640, 586, 524, 451, 362, 246, and 202 mAh g^−1^ at 0.1, 0.2, 0.5, 1.0, 2.0, 5.0, and 10.0 A g^−1^, respectively, considerably higher than those of the bare Fe_2_N-FBs and pure N-CFBs, revealing the excellent kinetics of Fe_2_N@N-CFBs. The outstanding rate capability and cycling stability of the Fe_2_N@N-CFBs electrode are further demonstrated in Fig. 4e. The Fe_2_N@N-CFBs electrode is also able to accommodate the continuously switched rate and cycling tests at repeated current densities. The quantitative kinetic analysis shows that the excellent rate performance originates mainly from the surface-controlled capacitive charge storage contribution by the 1D hierarchical fibrous structure of Fe_2_N@N-CFBs (Fig. S17). Figure 4f shows the prolonged cycling stabilities of Fe_2_N@N-CFBs at high current densities of 1.0 and 2.0 A g^−1^. Fe_2_N@N-CFBs delivers discharge capacities of 550 and 420 mAh g^−1^ after 1000 cycles at 1.0 and 2.0 A g^−1^, respectively. The EI tests indicate that the Fe_2_N@N-CFBs electrode exhibits a small impedance evolution during the repeated cycles (Fig. S18). These results show the structural durability of the robust multicore–shell Fe_2_N@N-CFBs.

**Fig. 5:**
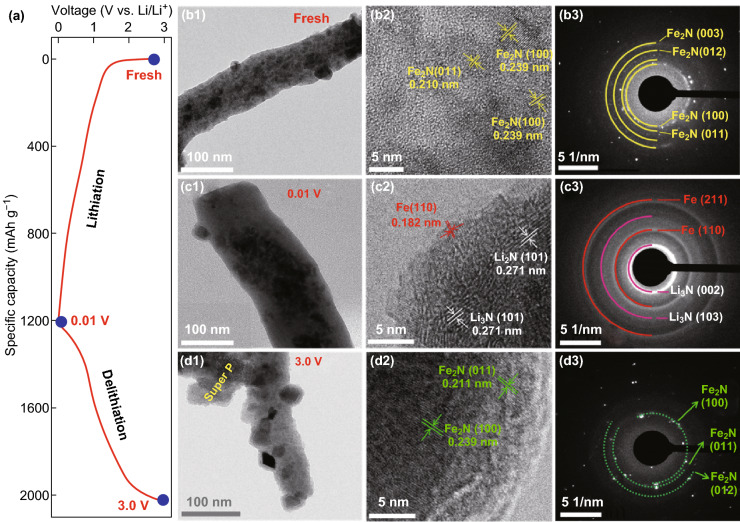
**a** Initial discharge/charge curves of Fe_2_N@N-CFBs at 50 mA g^−1^ in the voltage range of 0.01 −3.0 V. **b**–**d** TEM, HRTEM, and SAED images of the Fe_2_N@N-CFBs electrode recorded in the fresh **b1**–**3**, discharged **c1**–**3**, and charged **d1**–**3** states

To understand the electrochemical lithium storage mechanism of Fe_2_N@N-CFBs, ex situ TEM and HRTEM along with selected-area electron diffraction (SAED) were employed to analyze the phase evolution. Figure 5 shows TEM, HRTEM, and SAED images of the Fe_2_N@N-CFBs electrode recorded in the fresh, lithiated, and delithiated states in the voltage range of 0.01−3.0 V (Fig. 5a). The TEM image (Fig. 5b1) shows the multicore–shell structure of Fe_2_N@N-CFBs, while the HRTEM image (Fig. 5b2) and corresponding SAED pattern (Fig. 5b3) reveal the crystalline structure of the inner Fe_2_N NPs in the pristine Fe_2_N@N-CFBs. Upon discharge to 0.01 V, the lithiation of Fe_2_N@N-CFBs led to the pulverization of most of the Fe_2_N NPs and even disintegration into smaller particles, while the carbon nanofiber matrix inhibited their aggregation to some extent (Fig. 5c1). In this state, the (110) planes of Fe and (101) facet of Li_3_N can be observed in the HRTEM image (Fig. 5c2), while the diffraction rings in the SAED pattern (Fig. 5c3) confirm the presence of polycrystalline metallic iron (JCPDS No. 89-4185) and β-Li_3_N (JCPDS No. 78-2005), which indicates the conversion process for Fe_2_N NPs. Subsequently, upon the delithiation of the electrode in the range of 0.01–3.0 V, the conversion products change back to the Fe_2_N phase (Fig. 5d1–3), suggesting that the phase conversion reaction of Fe_2_N@N-CFBs is highly reversible. Ex situ XRD (Fig. S19) performed after discharging and charging further demonstrates the formation of crystalline Li_3_N and recovery of Fe_2_N. Based on the above results, the electrochemical lithium storage mechanism of Fe_2_N@N-CFBs may be explained by Fe_2_N + 3Li^+^ + 3e^−^ ↔ 2Fe + Li_3_N.

### Electrochemical Properties in Potassium-Ion Storage

**Fig. 6:**
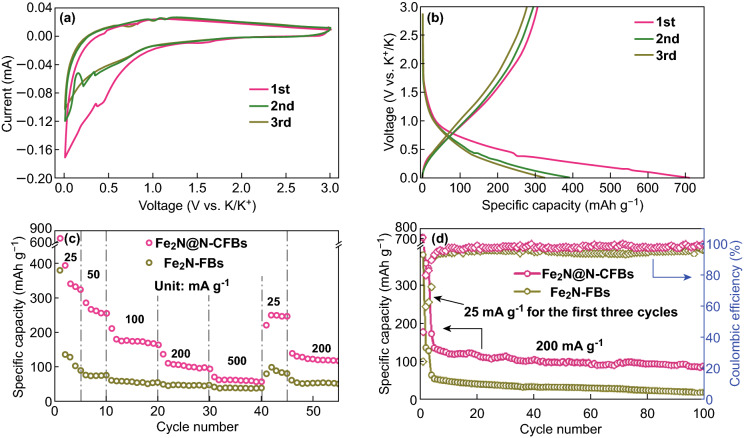
Potassium-ion storage properties of Fe_2_N@N-CFBs. **a** CV curves and **b** discharge–charge profiles at 25 mA g^−1^ for Fe_2_N@N-CFBs in the first three cycles. **c** Rate capabilities of the Fe_2_N@N-CFBs and Fe_2_N-FBs electrodes at varied current density. The presented capacities are discharge capacities. **d** Cycling performances of the Fe_2_N@N-CFBs and Fe_2_N-FBs electrodes for 100 cycles at 200 mA g^−1^

The potassium-ion storage properties of Fe_2_N@N-CFBs were also evaluated. Figure 6a shows the first three CV curves at a scan rate of 0.1 mV s^−1^ in the voltage range of 0.01–3.0 V (vs. K/K^+^). Only two typical peaks at 0.5 and 1.0 V are observed in the cathodic and anodic scans in the first cycle, which are associated with the potassiation and depotassiation of Fe_2_N, respectively. Similar results are observed in the CV tests of the bare Fe_2_N-FBs electrode (Fig. S20). The charge–discharge curves of Fe_2_N@N-CFBs at 25 mA g^−1^ are shown in Fig. 6b. The initial discharge and charge capacities are 710 and 310 mAh g^−1^, respectively, indicating an ICE of 43.7%. The low CE can be attributed to the formation of the SEI layer and irreversible insertion of potassium in the carbon defect sites of Fe_2_N@N-CFBs [48, 49]. In contrast, the Fe_2_N-FBs electrode exhibits considerably lower discharge and charge capacities of 379 and 92 mAh g^−1^, respectively, with a corresponding considerably lower ICE of 24.3% (Fig. S21). Similar to the LIB voltage profiles, the initial discharge/charge curves of the PIBs are also sloping and no obvious plateaus are observed, which can be attributed to the insufficient activation of the potassiation/depotassiation processes [50, 51]. Additionally, the reversible capacity of Fe_2_N@N-CFBs for potassium storage is considerably lower than that for lithium storage. The large radius of K^+^ compared to that of Li^+^ leads to a larger volume expansion and worse reaction kinetics during the discharge/charge process. The rate capacity of the Fe_2_N@N-CFBs electrode is shown in Fig. 6c. At the current densities of 25, 50, 100, 200, and 500 mA g^−1^, the reversible capacities of the composite are approximately 340, 242, 174, 110, and 85 mAh g^−1^, respectively. When the current density is returned to 25 mA g^−1^, the reversible capacity of 245 mAh g^−1^ can be retained and reaches approximately 125 mAh g^−1^ at 200 mA g^−1^ during the following cycles, demonstrating the highly reversible potassium-ion storage properties of Fe_2_N@N-CFBs. The cycling performance of the Fe_2_N@N-CFBs composite at 200 mA g^−1^ is shown in Fig. 6d. Although a small decrease in capacity is observed, the Fe_2_N@N-CFBs electrode maintains a reversible capacity of 102 mAh g^−1^ after 100 cycles. The CE is still close to 100% after the first few cycles. To the best of our knowledge, this is the first demonstration of iron nitride/carbon composites as anode materials for applications in PIBs. To confirm the structural merits of the multicore–shell Fe_2_N@N-CFBs, the cycling behavior of the Fe_2_N-FBs electrode was also evaluated for comparison under the same conditions (Fig. 6d). However, the bare Fe_2_N-FBs delivered a low reversible capacity of only 64 mAh g^−1^ in the first cycle and exhibited a gradual capacity fading in the subsequent cycles, leading to a negligible capacity as low as ≈ 22 mAh g^−1^ at the 100^th^ cycle. The relatively low reversible capacity of Fe_2_N-FBs is attributed mainly to the absence of elastic carbon framework protection during the continuous electrochemical cycling.

The cycling and rate performances of the 1D multicore–shell Fe_2_N@N-CFBs in LIBs are comparable to those of most reported TMN-based anode materials (Table S1). To better understand the excellent cycling behaviors, the electrode materials for LIBs after cycling (in the full-delithiation state) were further investigated by FESEM and TEM. As shown in Fig. S22a, b, the hierarchically fibrous structure of Fe_2_N@N-CFBs is well preserved, with a uniform and thin layer of SEI on the surface of the carbon fiber. The Fe_2_N NPs are homogeneously dispersed but still encapsulated in the carbon nanofiber framework without shedding or aggregation (Fig. S22c, d), suggesting that the carbon fiber framework in Fe_2_N@N-CFBs is sufficiently robust to accommodate the volume changes during the repeated charge–discharge process. Compared with the original Fe_2_N@N-CFBs (Fig. 1g, f), the particle size of Fe_2_N after the cycling is decreased with more expected nanograins, confirming the activation and electrochemical milling process during the lithiation/delithiation operation, well explaining the capacity increase upon the cycling. In contrast, the Fe_2_N-FBs electrode, as shown in Fig. S22e, f, not only suffers from a serious structural fracture owing to the internal strain caused by the considerable volume variation of the Fe_2_N particles, but also exhibits an extremely coarse and uneven surface, which could be associated with the thick and incomplete SEI layer. The electrode thicknesses before and after the cycling were also analyzed by FESEM (Fig. S23a–d). The Fe_2_N@N-CFBs electrode exhibits a considerably smaller increase in electrode average thickness (from 7.6 to 8.0 μm, ≈ 5.2%) than that of the bare Fe_2_N-FBs electrode (from 4.7 to 7.1 μm, ≈ 51%) after the 50th cycle discharging at 200 mA g^−1^ (lithiated state). We further used the volumetric capacity based on the lithiation-state electrode volume (highest thickness/volume during the cycling) to represent the electrochemical performance (see Experimental Section for the details of this calculation). The Fe_2_N@N-CFBs electrode can deliver a reversible volumetric capacity of 854 mAh cm^−3^ at the current density of 262 mA cm^−3^ after the 50th cycle (Fig. S23f), considerably higher than that of the bare Fe_2_N-FBs electrode (only 223 mAh cm^−3^).

**Fig. 7:**
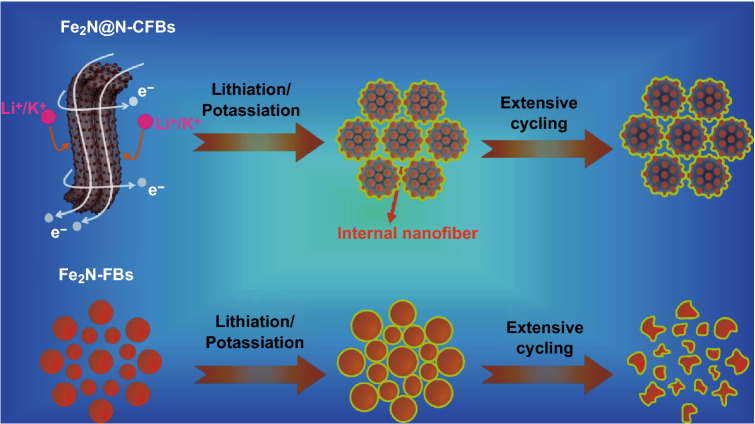
Schematic of the different structural evolutions of the Fe_2_N@N-CFBs (top panel) and Fe_2_N-FBs (bottom panel) electrode materials during the electrochemical lithium/potassium storage reaction. The light-yellow layer coating on the surface represents the SEI film. (Color figure online)

The remarkable electrochemical performance of Fe_2_N@N-CFBs can be attributed to the advantages of the 1D multicore–shell structure, as illustrated in Fig. 7. First, the all-around encapsulation architecture enables a large contact interface between the N-doped carbon nanofiber framework and Fe_2_N NPs along with continuous electron/ion transport pathways in both radial and axial directions, promoting the electrode reaction kinetics. In addition, the growth of the stable SEI layer on the surface of the carbon fiber prevents the continual rupturing and reformation of the SEI film, which commonly occur in all-alloy-based anode materials. Second, the multicore–shell architecture together with the long-range 1D carbon nanofiber framework can provide the elastic buffering function to accommodate the volume expansion/contraction of the impregnated Fe_2_N NPs. Third, the compact carbon nanofiber bundles are sufficiently robust to ensure the stable lithiation/potassiation of each internal Fe_2_N@carbon nanofiber contained inside N-CFBs, favoring the achievement of an excellent cycling stability during long-period cycles.

## Conclusion

We developed a viable biomass-based structural engineering strategy based on the use of SCFs for the template crafting of hierarchical multicore–shell Fe_2_N@N-CFBs as advanced anode materials for both LIBs and PIBs. These intriguing NP-encapsulated 1D conductive nanofiber bundle heterostructures not only retained the intrinsic advantages upon the Fe_2_N nanosizing, but also offered other benefits, such as the effective restraining of the volume variation of Fe_2_N and enhanced Fe_2_N conversion kinetics. Consequently, the Fe_2_N@N-CFBs composite achieved increased lithium-/potassium-ion storage performances. Furthermore, as the metal ions immobilized on the SCFs were optionally tunable, this facile approach utilizing the chemical reactivity and structural replicability of collagen fibers can be easily extended to the design and crafting of other advanced metal-nitride-based materials toward wider energy storage and conversion applications.

## Electronic supplementary material

Below is the link to the electronic supplementary material.

Supplementary material 1 (PDF 2487 kb)
