# Supplementary material for: Bio-Derived Hierarchical Multicore–Shell Fe2N-Nanoparticle-Impregnated N-Doped Carbon Nanofiber Bundles: A Host Material for Lithium-/Potassium-Ion Storage
Source: Nanomicro Lett. 2019 Jul 15;11:56. doi: 10.1007/s40820-019-0290-0 (PMC7770912; doi:10.1007/s40820-019-0290-0)
Supplement: Supplementary file 1 — Supplementary material 1 (PDF 2487 kb) [file 40820_2019_290_MOESM1_ESM.pdf]

Supporting Information for

Bio-derived Hierarchical Multicore–shell Fe_2N-Nanoparticle-Impregnated N-doped Carbon Nanofiber Bundles: A Host Material for Lithium-/Potassium-ion Storage

Hongjun Jiang[1, +], Ling Huang[1, +], Yunhong Wei[1], Boya Wang[1], Hao Wu[1, *], Yun Zhang[1, *] Huakun Liu[2], Shixue Dou[2]

[1]Department of Advanced Energy Materials, College of Materials Science and Engineering, Sichuan University, Chengdu, 610064, People's Republic of China

[2]Institute for Superconducting & Electronic Materials, Australian Institute of Innovative Materials, University of Wollongong, Wollongong, NSW 2500, Australia

[+]Hongjun Jiang and Ling Huang contributed equally to this work.

*Corresponding authors. E-mail address: hao.wu@scu.edu.cn (Hao Wu); y_zhang@scu.edu.cn (Yun Zhang)

Supporting Figures and Tables

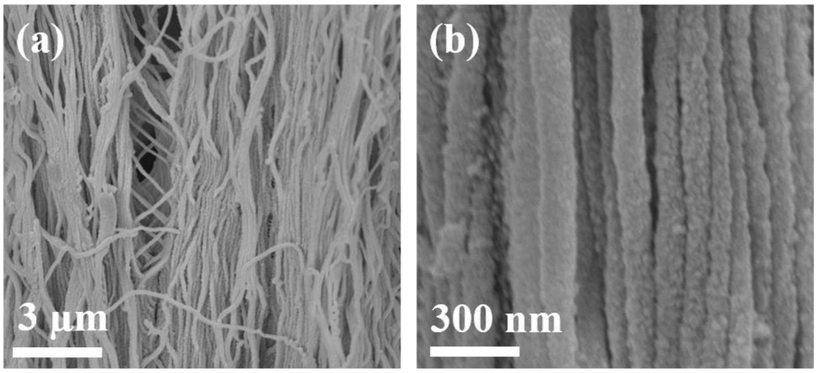

Fig. S1 FESEM images of SCFs@Fe^{3+} intermediates at different magnifications

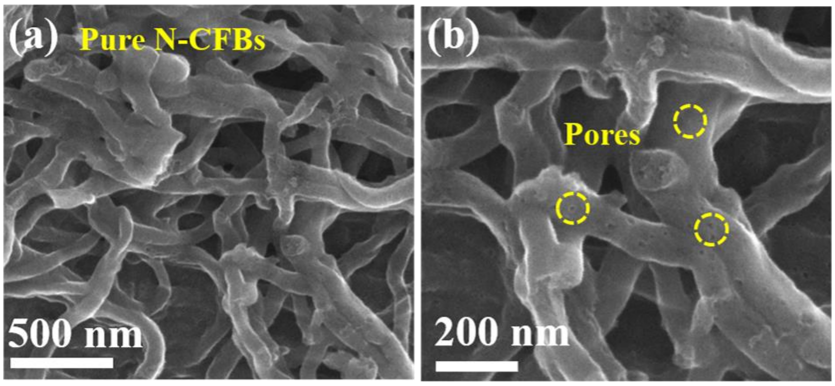

Fig. S2 FESEM images of pure N-CFBs

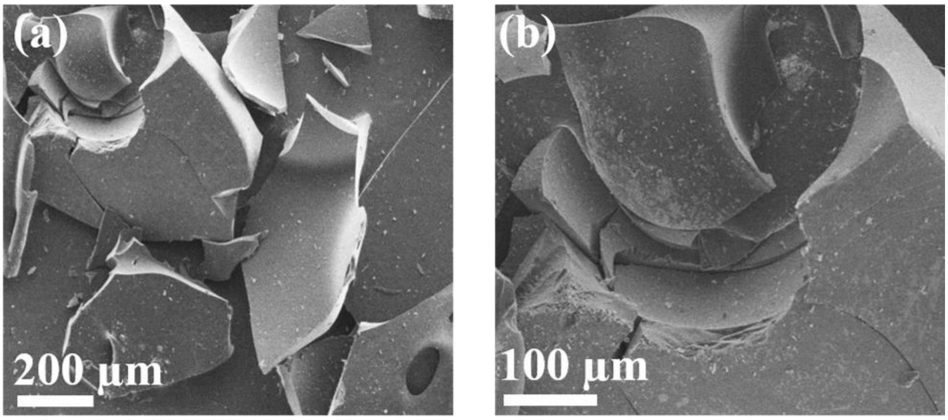

Fig. S3 FESEM images of carbonized skin collagen fibers by directly calcining in NH_3 atmosphere at 500 °C

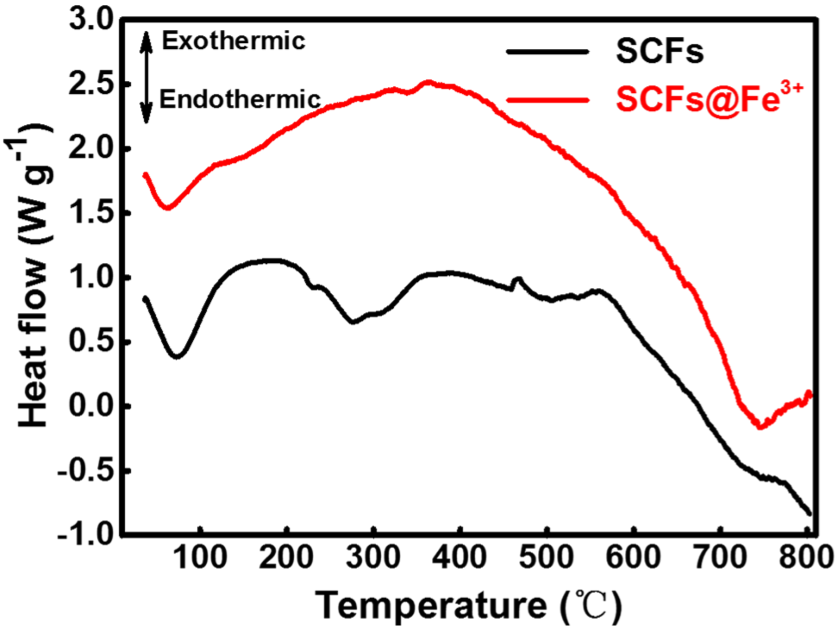

Fig. S4 Differential scanning calorimetry (DSC) curves of native skin collagen fibers (SCFs) and Fe^{3+}-immobilized SCFs (SCFs@Fe^{3+}) in Ar atmosphere at a heating rate of 10 °C min^{-1}

As shown in **Fig.** S4, the Fe^{3+}-immobilized skin collagen fibers (SCFs@Fe^{3+}) show remarakly enhanced thermal stability with a higher decomposition temperature (≈370 °C) than that of pristine SCFs (≈175 °C). This result further confirms that the complexing interaction of SCFs with iron salts is indeed able to increase the degree of cross-linking between the collagen macromolecules, as well as significantly improving the thermal shrinkage temperature of SCFs.

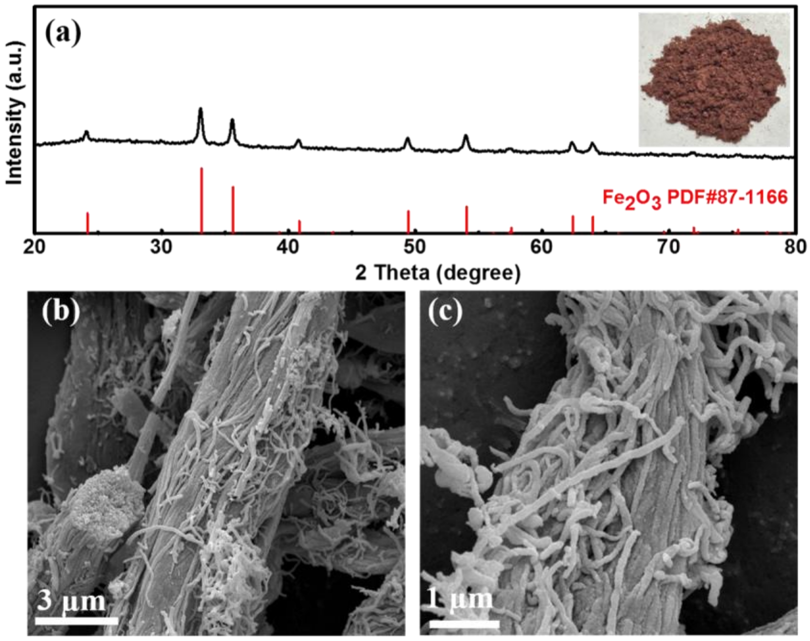

Fig. S5 a XRD pattern and **b, c** FESEM images of Fe_2O_3 prepared by calcinating the SCFs@Fe^{3+} in air. The inset in **a** is a photograph of the resultant Fe_2O_3 powder

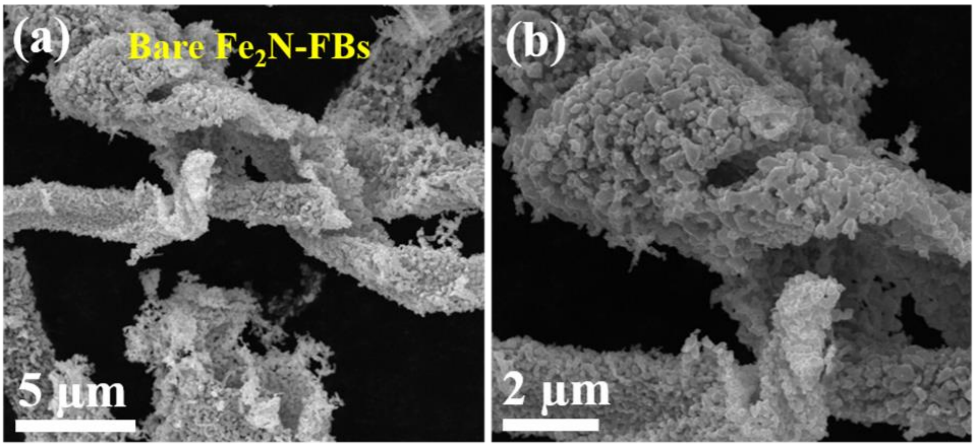

Fig. S6 FESEM images of bare Fe_2N-FBs

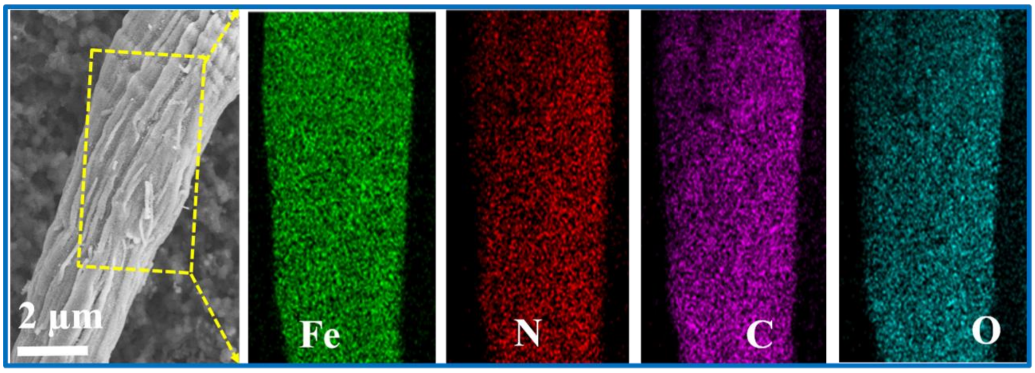

Fig. S7 SEM-EDS mapping images of the Fe_2N@N-CFBs

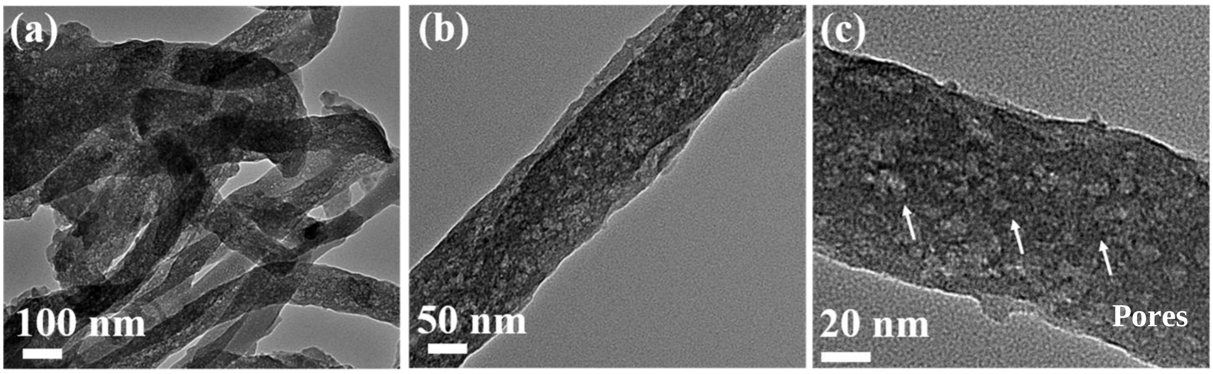

Fig. S8 TEM images of pure N-CFBs

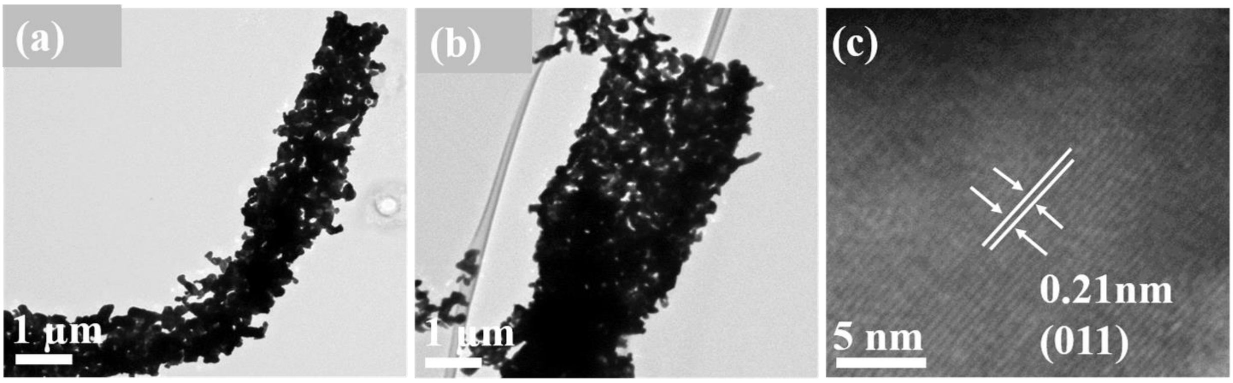

Fig. S9 TEM images of bare Fe_2N-FBs

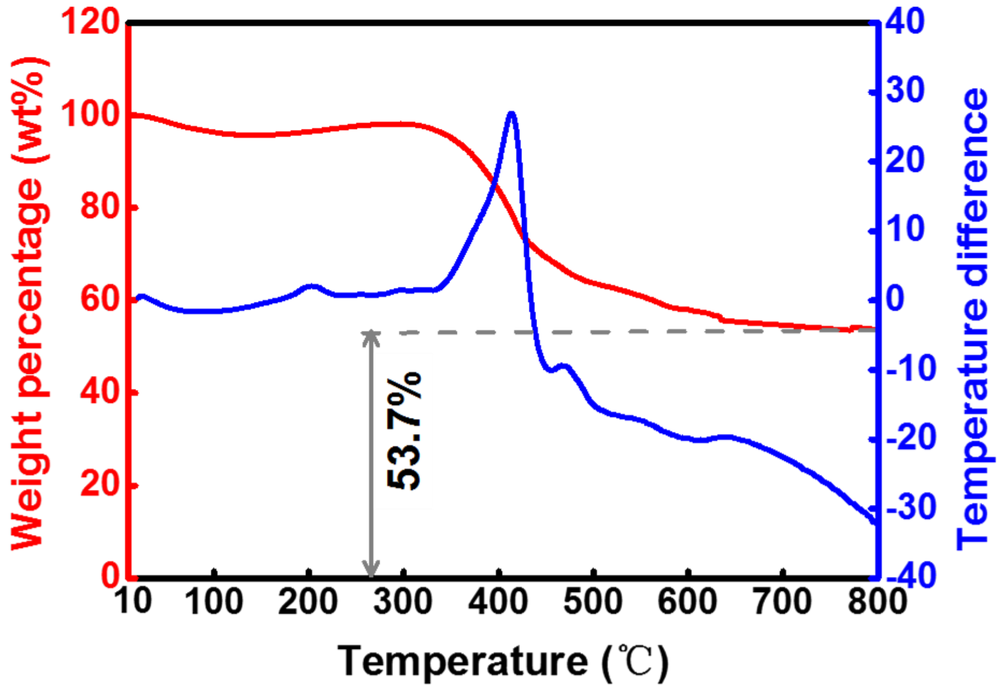

Fig. S10 TGA and differential thermogravimetry (DTG) curves of Fe_2N@N-CFBs collected in air atmosphere at a heating rate of 10 °C min^{-1}

As shown in **Fig.** S10, the weight loss below 200 °C is mainly due to the loss of absorbed gaseous molecules. An obvious weight increase between 200 and 300 °C is related to the oxidation of

external Fe_2N. The sharp decrease in weight between 350 and 400 °C is mainly associated with the burning of N-CFBs. A relatively slow weight loss between 400 and 700 °C is attributed to two processes, the combustion of N-CFBs and oxidation of Fe_2N, in which the transformation from Fe_2N to Fe_2O_3 gives a weight increase of 27%. Considering that the Fe:N ratio is 2:0.84, we assume the $Fe_2N_{0.84}$ content is x, and therefore, the weight content of $Fe_2N_{0.84}$ can be calculated using the following formula:

$$x \times \frac{112}{112 + 14 \times 0.84} = 53.7\% \times \frac{112}{160}$$

Thus, the Fe_2N content is 41.5%.

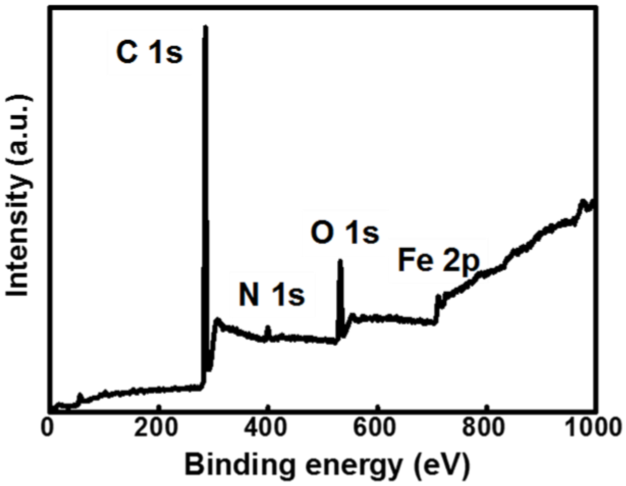

Fig. S11 XPS survey scan of Fe_2N@N-CFBs

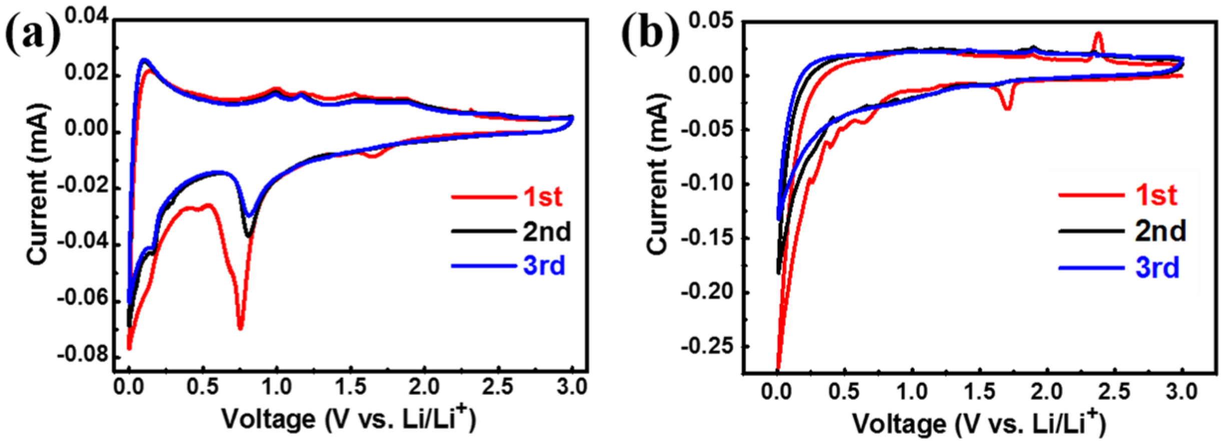

Fig. S12 CV curves of **a** bare Fe_2N-FBs and **b** pure N-CFBs at a scan rate of 0.1 mV s^{-1}

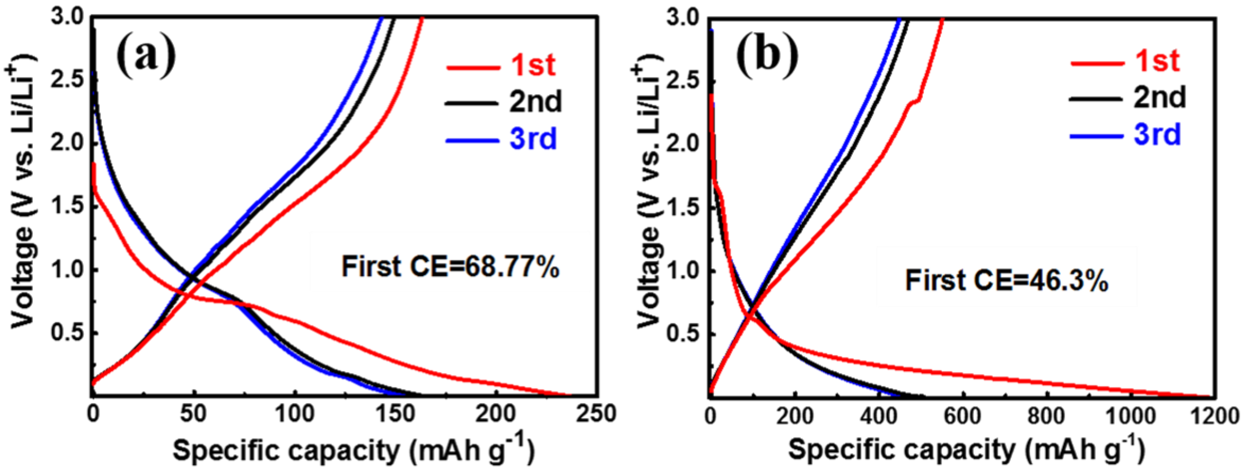

Fig. S13 Discharge-charge curves of **a** bare Fe_2N-FBs and **b** pure N-CFBs at the current density of 0.1 A g^{-1}

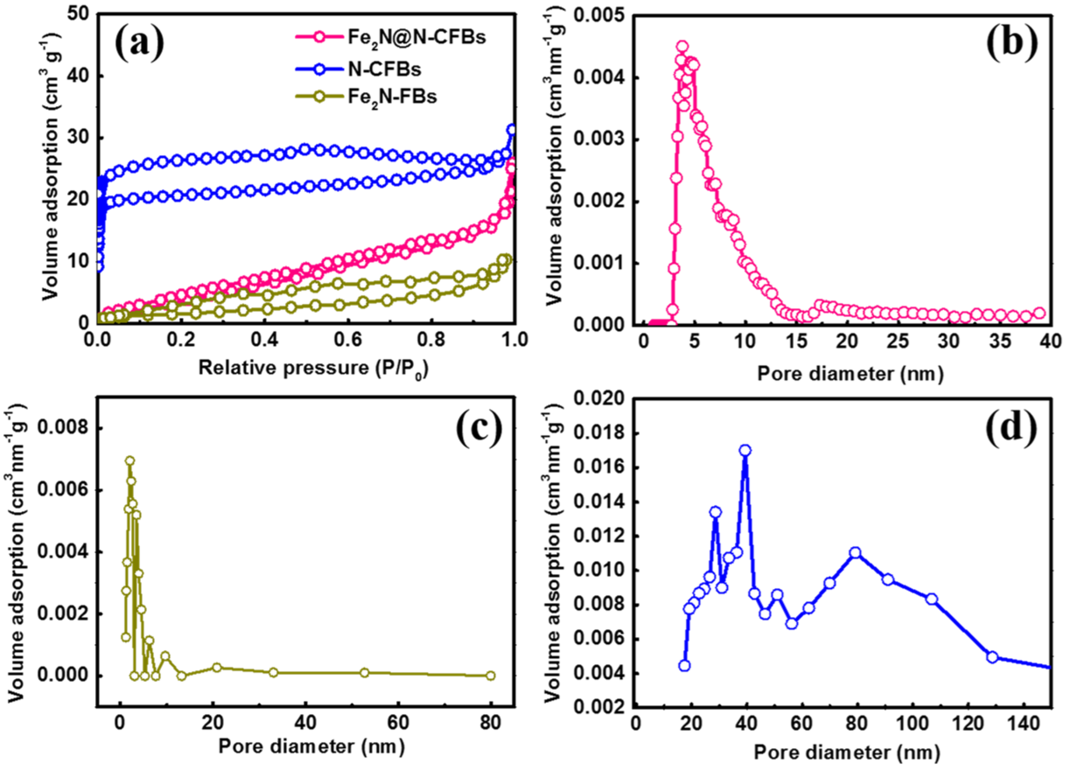

Fig. S14 a N_2 sorption/desoprtion isotherm of the Fe_2N@N-CFBs, Fe_2N-FBs, and pure N-CFBs electrodes. **b-d** Pore size distribution of the Fe_2N@N-CFBs, Fe_2N-FBs, and pure N-CFBs electrode

As shown in **Fig.** S14a, the Brunauer-Emmett-Teller (BET) specific surface of the Fe_2N@N-CFBs measured here is 20.8 m^2 g^{-1}, much higher than that of Fe_2N-FBs (6.8 m^2 g^{-1}), but significantly lower than the value for pure N-CFBs (57.2 m^2 g^{-1}). Pore size distribution demonstrates that mesopores dominate in the Fe_2N@N-CFBs and Fe_2N-FBs electrodes evidently, whereas a significant amount of macro-pores are observed in the pure N-CFBs electrode, as displayed in **Fig.** S14b-d. The presence of macro-pores is resulted from the removal of Fe_2N nanoparticles. The much higher surface area of pure N-CFBs could result in lower ICE because of the inevitable surficial chemisorption of Li ions into the pores as well as defect sites of carbon fibers, which is likely to

cause more irreversible lithium-ion consumption. Whilst electrode materials with small surface area, for instance, 6.8 m^2 g^{-1} for Fe_2N-FBs, is unfavored for the rapid transportation of Li ions and the wettability of electrolyte to the eletrode surface, the Fe_2N@N-CFBs with an appropriate surface area of 20.8 m^2 g^{-1} is able to provide sufficient active sites for surface chemisorption as well as rapid electrolyte and Li ion diffusion passages, and thus achieving a relatively high ICE of 73.5%.

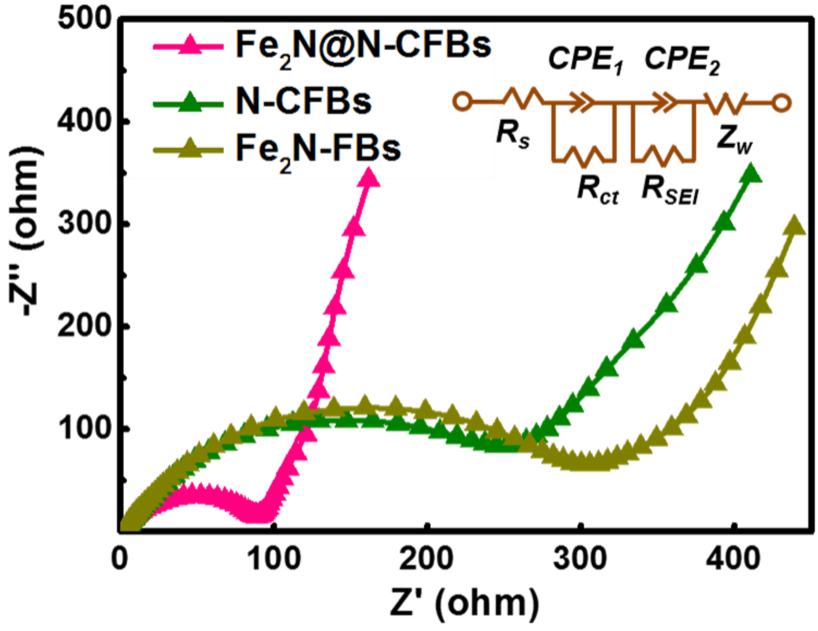

Fig. S15 EIS spectra of Fe_2N-FBs and Fe_2N@N-CFBs with the equivalent circuit in the inset

As shown above, the EIS profiles for both the Fe_2N@N-CFBs and Fe_2N-FBs electrodes show a compressed semicircle from the high to medium frequency range of each spectrum and an inclined line in the low-frequency range. The diameter of the compressed semicircle is ascribed to the charge transfer resistance (R_{ct}) for these electrodes, and the inclined line is considered to be related to the Warburg impedance (Z_w). In the equivalent circuit, R_s represents the Ohmic resistance of the electrode system, including the electrolyte and the cell components. R_{SEI} is the resistance due to the SEI. R_{ct} represents the resistance related to charge transfer, and the constant phase element (CPE) reflects the double-layer capacitance. Z_w represents the Warburg impedance, which is attributed to the Li-ion diffusion in the solid. Apparently, the ohmic resistance (R_s) values of the two electrodes are almost the same, but the Fe_2N@N-CFBs one exhibits a lower R_{ct} of ≈90 Ω. This implies that the Fe_2N@N-CFBs features better electrical contact and a faster charge transfer reaction for Li-ion insertion/extraction compared with the bare Fe_2N-FBs.

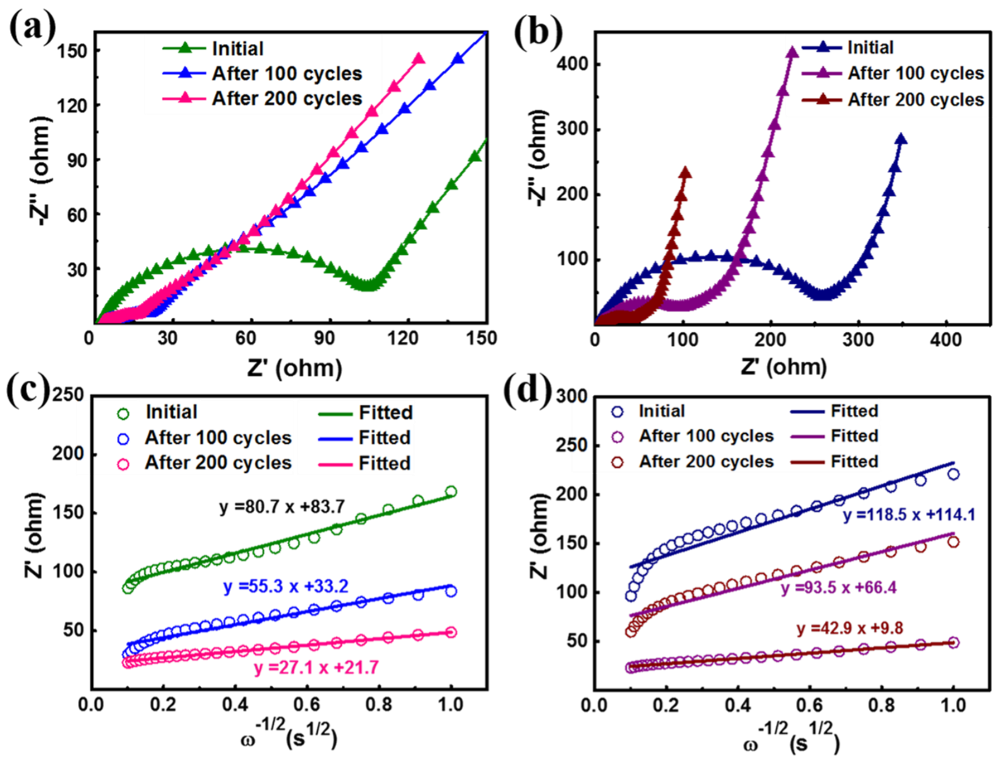

Fig. S16 Electrochemical impedance spectra of (a) Fe_2N@N-CFBs and (b) bare Fe_2N-FBs collected during the cycling test at 0.2 A g^{-1}; The corresponding linear fitted curves of Z'versus $\omega^{-1/2}$ of the fresh (c) Fe_2N@N-CFBs and (d) Fe_2N-FBs electrodes and after the 100th and 200th cycles..

Figure S16 presents the EIS curves of Fe_2N@N-CFBs and Fe_2N-FBs electrodes after different numbers of cycles at 0.2 A g^{-1}. As seen in **Fig.** S16a, the R_{ct} for Fe_2N@N-CFBs dramatically decreases to 30 and 25 Ω after 100 and 200 cycles, respectively. Note that the reduction in R_{ct} for the Fe_2N@N-CFBs is more significant than that for the bare Fe_2N-FBs (100 Ω and 50 Ω, **Fig.** S16b), confirming that the Fe_2N NPs encapsulated in the highly electrically conducting 1D N-doped carbon nanofiber framework are capable of greatly enhanced rapid electron transfer during the Li^+ insertion/extraction process. In addition, the lithium-ion diffusion coefficient (D_{Li}, cm^2 s^{-1}) can be calculated from the inclined lines in the Warburg region using Eqs. S1 and S2 [S1, S2]:

$$Z' = R + \sigma\omega^{-1/2} \qquad \text{(S1)}$$

$$D = (R^2T^2)/(2A^2n^4F^4C^2\sigma^2) \qquad \text{(S2)}$$

In these equations, ω is the angular frequency, R is the universal gas constant, T is the absolute temperature, A is the active surface area of the electrode, n is the number of electrons, F is the Faraday constant, and C is the concentration of lithium ions. σ is the Warburg factor which could be calculated from the gradient of the oblique line Z'–$\omega^{-1/2}$ ($\omega = 2\pi f$) in the low-frequency region. As displayed in **Fig.** S16c-d, the slope of the straight lines for both the Fe_2N@N-CFBs and the Fe_2N-FBs have remarkably decreased after cycling, although the Fe_2N@N-CFBs exhibits a rather lower slope than that of the bare Fe_2N-FBs, indicating its faster Li^+ diffusion process. The calculated D_{Li} of Fe_2N@N-CFBs after 200 cycles is 1.60×10^{-17} cm^2 s^{-1}, which is over 2.5 times that of Fe_2N-FBs (6.36×10^{-18} cm^2 s^{-1}). Such high performance for Fe_2N@N-CFBs could be ascribed to the SCF-derived oxygen-containing carbon nanofiber backbone together with the

nitrogen heteroatom doping. These factors significantly boost the electrical conductivity and the surface wettability of electrode, which allows efficient contact of the embedded Fe_2N NPs with the electrolyte, and results in faster electrode/electrolyte interface kinetics.

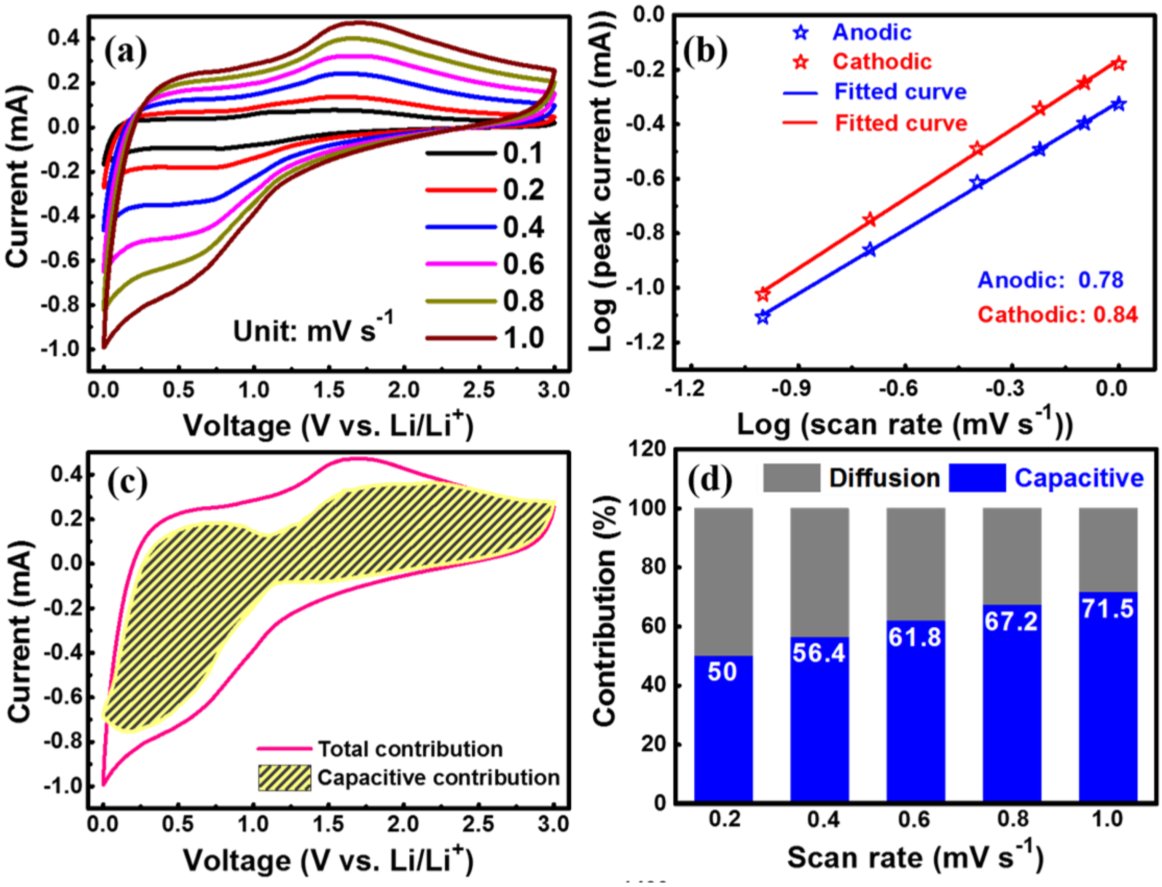

Fig. S17 a CV curves of Fe_2N@N-CFBs at different scan rates from 0.1 to 1.0 mV s^{-1}. **b** Plots of peak current versus the corresponding scan rate; **c** The CV curve at 1.0 mV s^{-1} with an estimated capacitive contribution in the yellow-shaded region. **d** The capacitive contribution ratio at various scan rates

The capacitive behavior was characterized by CV measurements at varied scan rates from 0.1 to 1.0 mV s^{-1}. In **Fig.** S17a, the CV curves of Fe_2N@N-CFBs electrode show a well-preserved shape along with steadily enhanced redox peaks as the scan rate increases from 0.1 to 1.0 mV s^{-1}. The peak currents (i) in different CV curves obey a power-law relationship with the scan rate (v) according to the following equation [S3, S4]: $i = av^b$, where a and b are parameters. By plotting log(i) versus log(v), the b value can be obtained from the slope of the profile. Specifically, the b value of 1 suggests a surface diffusion process (capacitor-type), while 0.5 reveals a totally diffusion-controlled behavior (battery-type). The plots for both the anodic and the cathodic peaks are shown in **Fig.** S17b. At scan rates ranging from 0.1 to 1.0 mV s^{-1}, the b values of the anodic and cathodic peaks are 0.78 and 0.84, respectively, which implies that the charge storage of Fe_2N@N-CFBs is mostly dominated by capacitive behavior. Thus, to further determine the potential regions where the capacitive contributions occur in the CV plots, the current is divided into capacitive effect and diffusion behavior through Eqs. S3 and S4 [S5]:

$$i = k_1 v + k_2 v^{1/2} \quad \text{(S3)}$$

$$i(V)/v^{1/2} = k_1 v^{1/2} + k_2 \qquad \text{(S4)}$$

Accordingly, the portion of the current from surface capacitance and diffusion contributions can be differentiated by determining both the k_1 and k_2 constants. Figure S17c shows the original CV curve of the total current and the calculated CV curve of the capacitive current (yellow-shaded section). Based on the integration of the CV curve, over 71% of the total charge storage of Fe_2N@N-CFBs electrode is capacitive at the scan rate of 1.0 mV s^{-1}. The percentages at other scan rates were also calculated as shown in **Fig.** S17d, and it displays that the capacitive capacity contribution of Fe_2N@N-CFBs electrode is gradually improving from 50 to 71.5% with the increase of scan rates from 0.2 to 1.0 mV s^{-1}. Hence, the well-defined capacitive behavior for the Fe_2N@N-CFBs electrode is attributed to the unique 1D hierarchically ordered fibrous framework that is organized from numerous staggered N-doped carbon nanofibers with uniformly dispersed Fe_2N NPs, which not only provides sufficient active sites for surface electrochemical reaction, but also creates direct and continuous transport pathways in both the radial and axial directions for Li ion diffusion, thereby enhancing the high-rate Li-ion storage capability.

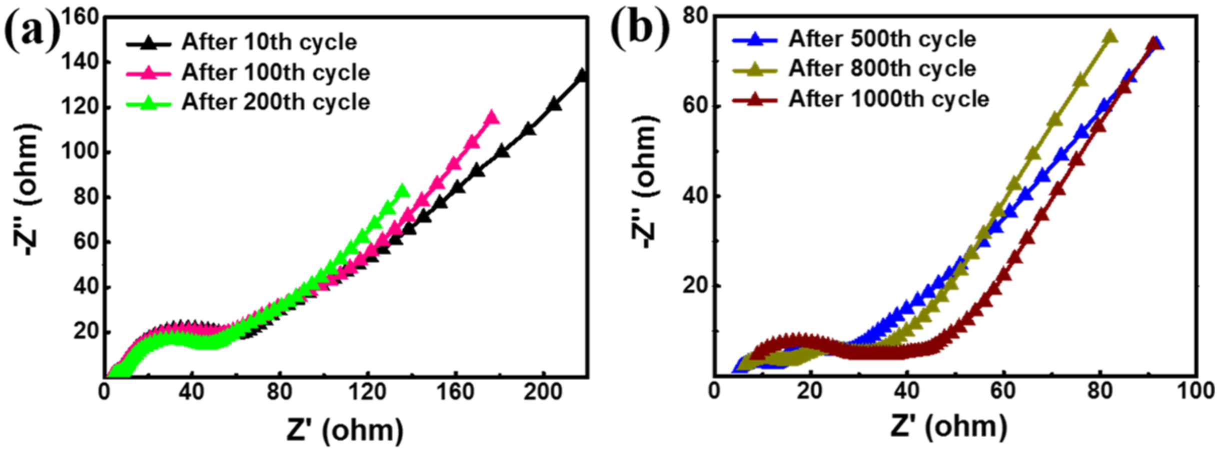

Fig. S18 a, b Electrochemical impedance spectra of Fe_2N@N-CFBs electrode collected during the cycling test at 1.0 A g^{-1}

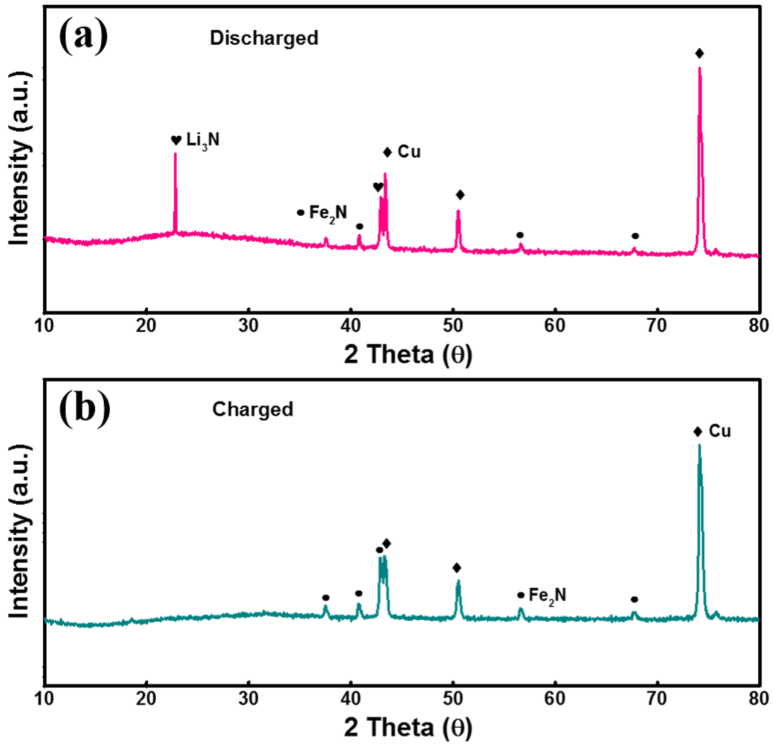

Fig. S19 Ex-situ XRD patterns of the Fe_2N@N-CFBs electrode after **a** discharging and **b** charging at 50 mA g^{-1} in the voltage range of 0.01−3.0 V

As shown in **Fig.** S19, from the XRD patterns, we observed metallic Cu, crystalline Li_3N, and Fe_2N. The strong signal of Cu comes from the current collector of copper foil. Peaks assigned to Li_3N confirms the formation of crystal products after discharging, consistent with the TEM results (**Fig.** 5c1-c3). After charging, all the peaks assigning to Li_3N disappeared, with only crystal Fe_2N and Cu detected, demonstrating that Fe_2N could react with Li^+ reversibly by generating Li_3N during charging/discharging process. It should be mentioned that the expected metallic Fe is not probed whilst Fe_2N crystal is still observable after discharging. The possible reason for the missing of Fe peaks could be due to the formation of small size Fe nanoparticles which shows low peak intensity. Also limited by our testing condition, the ex-situ XRD was carried out without protection, making it possible to form an amorphous oxidation layer on the surface of iron before testing, and thus could further lower the peak intensity and resulting in the burial of iron peaks underneath the strong Cu peaks. The detectable Fe_2N after discharging suggests incomplete conversion of Fe_2N, consistent with our argument of delayed wettability of eletrolyte to the inner Fe_2N nanoparticles.

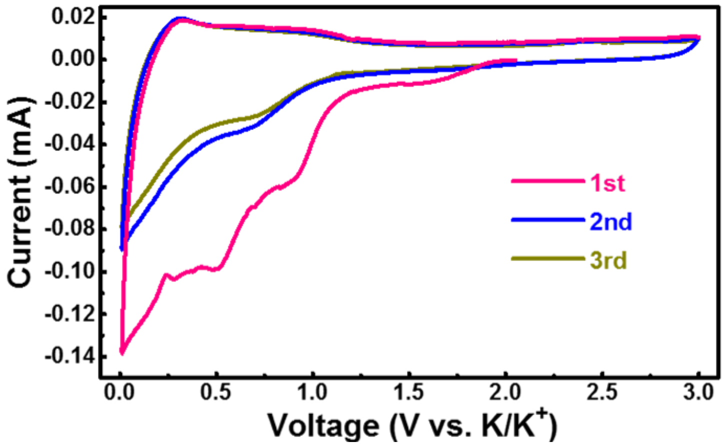

Fig. S20 CV curves of bare Fe_2N-FBs electrode at a scan rate of 0.1 mV s^{-1} in the first three cycles for PIBs

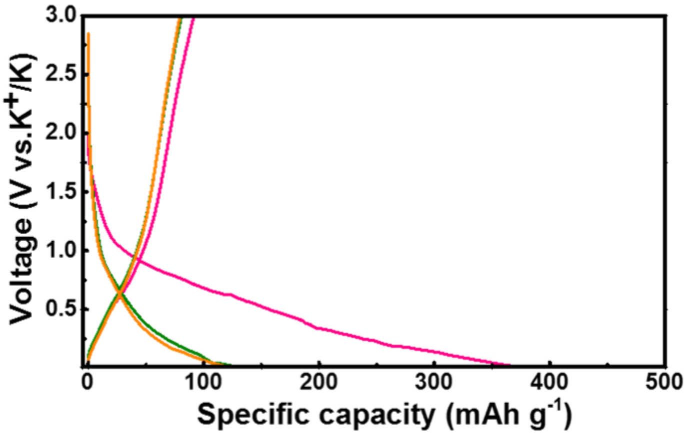

Fig. S21 Discharge-charge curves of bare Fe_2N-FBs electrode at the current density of 25 mA g^{-1} in the first three cycles for PIBs

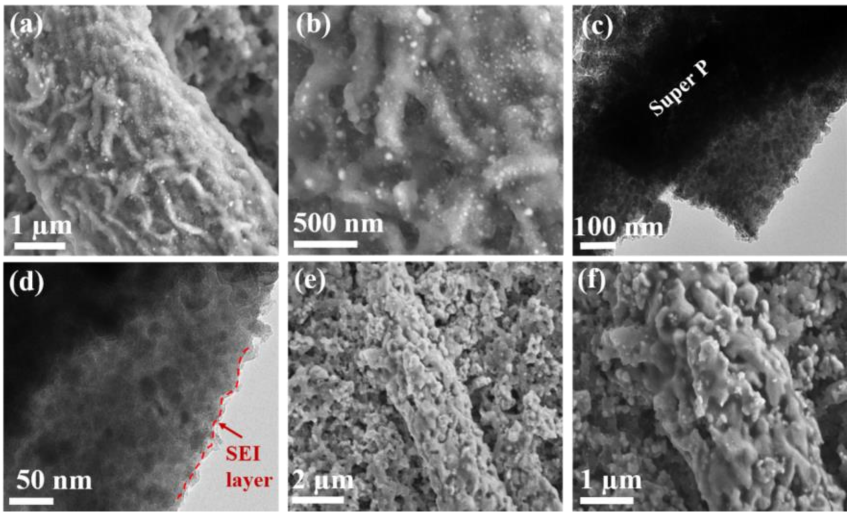

Fig. S22 a, b FESEM images and **c, d** TEM images of Fe_2N@N-CFBs electrode after 100 cycles at 0.5 A g^{-1}; **e, f** FESEM images of Fe_2N-FBs electrode after 100 cycles at 0.5 A g^{-1}

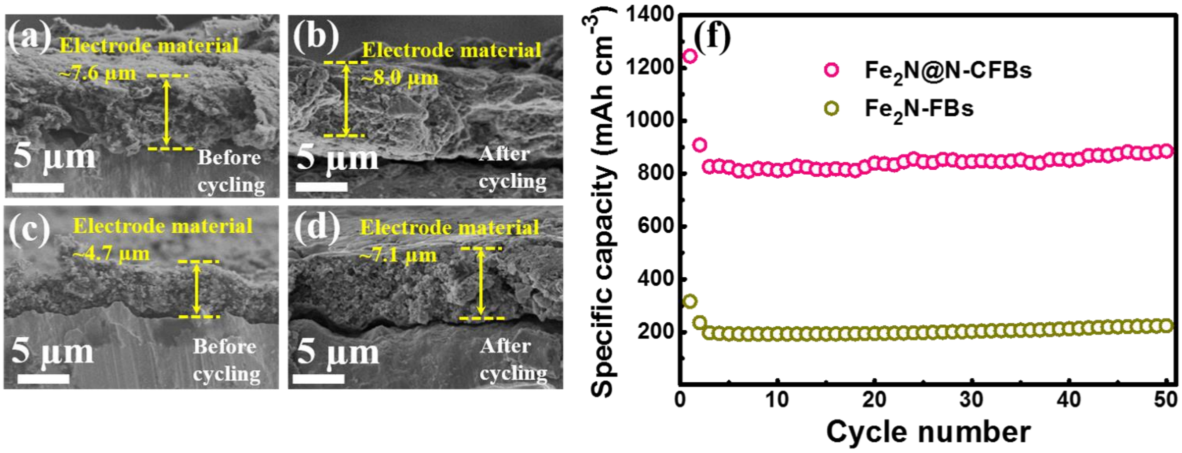

Fig. S23 FESEM images of cross-section of the **a, b** Fe_2N@N-CFBs and **c, d** Fe_2N-FBs electrodes before and after 50 cycles at 0.2 A g^{-1}. **f** The volumetric capacities of Fe_2N@N-CFBs and Fe_2N-FBs during cycling at a current density of 262 mA cm^{-3}

Table S1 Comparison of the electrochemical performance of previously reported metal nitride-based LIB anode materials with our work.

Electrode materials	Current density (A g^{-1})	2nd cycle capacity (mAh g^{-1})	Cycle number	Capacity retention (vs. 2nd cycle capacity)	References
**Multicore-shell**	**1.0**	**402**	**1000**	**144.3%**	**This work**
**Fe_2N@N-CFBs**	**2.0**	**321**	**1000**	**130.8%**	
Fe_3N@C	0.1	540	500	68.5%	[S6]
FeN/N-rG-O	0.1	510	100	78.1%	[S7]
Sn_3N_4	0.2	≈800	50	46.3%	[S8]
CrN	1.218	≈1050	200	68.9%	[S9]
Fe_2N@C-RGO	0.1	≈810	100	93.9%	[S10]
3D Ni_3N nanosheets	0.423	≈510	50	113.7%	[S11]
Porous Fe_2N@C	0.1	618	50	107.0%	[S12]
	10.0	391	2000	85.0%	
TiN-900	0.335	567	100	93.0%	[S13]
	1.675	288	100	93.0%	
MoN_x film	0.1 (μA cm^{-2})	≈900	100	77.3%	[S14]
Fe_2N/carbon textile	1.0	≈820	681	83.0%	[S15]
	6.0	≈310	300	77.4%	
Mn_3N_2	0.08	579	110	80.0%	[S16]
	0.16	500	300	75.1%	
VN	0.1	455	100	114.3%	[S17]
	1.0	341	250	75.0%	
Cu_3N	1.0	270	140	120.0%	[S18]

Table S2 Comparison of the electrochemical performance of the state-of-art electrode for PIBs with our work

Electrode materials	Current density (mA g^{-1})	2nd cycle capacity (mAh g^{-1})	Cycle number	Capacity retention (vs. 2nd cycle capacity)	References
**Multicore-shell Fe_2N@N-CFBs**	**200**	**≈125**	**100**	**82.6%**	**This work**
F-doped graphene foam	500	≈250	200	66.4%	[S19]
N-doped graphene	100	≈260	100	80.7%	[S20]
Amorphous orderd mesoporous carbon	200	≈270	200	73.0%	[S21]
VN quantum dot@N-doped carbon microsheets	100	≈325	100	76.9%	[S22]
Bamboo-like MnS_2/N-doped-C	100	≈350	200	60.6%	[S23]
Pure MnS_2	100	≈320	200	47.5%	
FeP	100	≈180	300	27.8%	[S24]
Zn@nanoporous carbon network	100	≈260	100	80.8%	[S25]
N-doped porous carbon derived from MOFs	2000	≈320	2000	72.2%	[S26]
Hard carbon	100	≈275	50	90.5%	[S27]
CoP/N,P-codoped porous carbon sheets	100	≈200	1000	63.5%	[S28]
$CoSe_2$@N-doped CNTs	200	≈350	100	81.4%	[S29]

Supplementary References

[S1] K.V. Sankar, R.K. Selvan, The ternary $MnFe_2O_4$/graphene/polyaniline hybrid composite as negative electrode for supercapacitors. J. Power Sources **275**, 399-407 (2015). https://doi.org/10.1016/j.jpowsour.2014.10.183

[S2] P. Ge, H. Hou, S. Li, L. Yang, X. Ji, Tailoring rod-like $FeSe_2$ coated with nitrogen-doped carbon for high-performance sodium storage. Adv. Funct. Mater. **28**(30), 1801765-1801776 (2018). https://doi.org/10.1002/adfm.201801765

[S3] P. Simon, Y. Gogotsi, B. Dunn, Where do batteries end and supercapacitors begin? Science **343**(6176), 1210-1211 (2014). https://doi.org/10.1126/science.1249625

[S4] J. Qian, F. Wu, Y. Ye, M. Zhang, Y. Huang et al., Boosting fast sodium storage of a large-scalable carbon anode with an ultralong cycle life. Adv. Energy Mater. **8**(16), 1703159-1703166 (2018). https://doi.org/10.1002/aenm.201703159

[S5] J. Wang, J. Polleux, J. Lim, B. Dunn, Pseudocapacitive contributions to electrochemical energy storage in TiO_2 (anatase) nanoparticles. J. Phy. Chem. C **111**(40), 14925-14931 (2007). https://doi.org/10.1021/jp074464w

[S6] H. Huang, S. Gao, A.M. Wu, K. Cheng, X.N. Li et al., Fe_3N constrained inside C nanocages as an anode for Li-ion batteries through post-synthesis nitridation. Nano Energy **31**, 74-83 (2017). https://doi.org/10.1016/j.nanoen.2016.10.059

[S7] L. Lai, J. Zhu, B. Li, Y. Zhen, Z. Shen et al., One novel and universal method to prepare transition metal nitrides doped graphene anodes for Li-ion battery. Electrochim. Acta **134**, 28-34 (2014). https://doi.org/10.1016/j.electacta.2014.04.073

[S8] X. Li, A.L. Hector, J.R. Owen, S.I.U. Shah, Evaluation of nanocrystalline Sn_3N_4 derived from ammonolysis of $Sn(NEt_2)_4$ as a negative electrode material for Li-ion and Na-ion batteries. J. Mater. Chem. A **4**(14), 5081-5087 (2016). https://doi.org/10.1039/C5TA08287K

[S9] I. Memona, A.S. Mustansar, A.U. Rehman, A. Nisar, M.M. Waheed et al., Mechanistic insights into high lithium storage performance of mesoporous chromium nitride anchored on nitrogen-doped carbon nanotubes. Chem. Eng. J. **327**, 361-370 (2017). https://doi.org/10.1016/j.cej.2017.06.095

[S10] P. Yu, L. Wang, F. Sun, D. Zhao, C. Tian et al., Three-dimensional Fe_2N@C microspheres grown on reduced graphite oxide for lithium-ion batteries and the Li storage mechanism. Chemistry **21**(8), 3249-3256 (2015). https://doi.org/10.1002/chem.201406188

[S11] M.S. Balogun, Y. Zeng, W. Qiu, Y. Luo, A. Onasanya et al., Three-dimensional nickel nitride (Ni3N) nanosheets: free standing and flexible electrodes for lithium ion batteries and supercapacitors. J. Mater. Chem. A **4**(25), 9844-9849 (2016). https://doi.org/10.1039/C6TA02492K

[S12] Y. Dong, B. Wang, K. Zhao, Y. Yu, X. Wang et al., Air-stable porous Fe2N encapsulated in carbon microboxes with high volumetric lithium storage capacity and a long cycle life. Nano Lett. **17**(9), 5740-5746 (2017). https://doi.org/10.1021/acs.nanolett.7b02698

[S13] M.S. Balogun, M. Yu, C. Li, T. Zhai, Y. Liu et al., Facile synthesis of titanium nitride nanowires on carbon fabric for flexible and high-rate lithium ion batteries. J. Mater. Chem. A **2**(28), 10825-10829 (2014). https://doi.org/10.1039/C4TA00987H

[S14] D.K. Nandi, U.K. Sen, D. Choudhury, S. Mitra, S.K. Sarkar, Atomic layer deposited molybdenum nitride thin film: a promising anode material for Li ion batteries. ACS Appl. Mater. Interfaces **6**(9), 6606-6615 (2014). https://doi.org/10.1021/am500285d

[S15] M.S. Balogun, M. Yu, Y. Huang, C. Li, P. Fang et al., Binder-free Fe_2N nanoparticles on carbon textile with high power density as novel anode for high-performance flexible lithium ion batteries. Nano Energy **11**, 348-355 (2015). https://doi.org/10.1016/j.nanoen.2014.11.019

[S16] Q. Sun, Z.W. Fu, Mn_3N_2 as a novel negative electrode material for rechargeable lithium batteries. Appl. Surf. Sci. **258**(7), 3197-3201 (2012). https://doi.org/10.1016/j.apsusc.2011.11.063

[S17] X. Peng, W. Li, L. Wang, L.S. Hu, W.H. Jin et al., Lithiation kinetics in high-performance porous vanadium nitride nanosheet anode. Electrochim. Acta **214**, 201-207 (2016). https://doi.org/10.1016/j.electacta.2016.08.023

[S18] R. Deshmukh, G. Zeng, E. Tervoort, M. Staniuk, D. Wood et al., Ultrasmall Cu_3N nanoparticles: surfactant-free solution-phase synthesis, nitridation mechanism, and application for lithium storage. Chem. Mater. **27**(24), 8282-8288 (2015). https://doi.org/10.1021/acs.chemmater.5b03444

[S19] Z. Ju, S. Zhang, Z. Xing, Q. Zhuang, Y. Qiang et al., Direct Synthesis of few-layer F-doped graphene foam and its lithium/potassium storage properties. ACS Appl. Mater. Interfaces **8**(32), 20682-20690 (2016). https://doi.org/10.1021/acsami.6b04763

[S20] Keith Share, Adam P. Cohn, Rachel Carter, Bridget Rogers, C.L. Pint, Role of nitrogen-doped graphene for improved high-capacity potassium ion battery anodes. ACS Nano **10**(10), 9738-9744 (2016). https://doi.org/10.1021/acsnano.6b05998

[S21] W. Wang, J. Zhou, Z. Wang, L. Zhao, P. Li et al., Short-range order in mesoporous carbon boosts potassium-ion battery performance. Adv. Energy Mater. **8**(5), 1701648 (2018). https://doi.org/10.1002/aenm.201701648

[S22] H. Wu, Q. Yu, C.-Y. Lao, M. Qin, W. Wang et al., Scalable synthesis of VN quantum dots encapsulated in ultralarge pillared N-doped mesoporous carbon microsheets for superior potassium storage. Energy Storage Mater. **18**, 43-50 (2018). https://doi.org/10.1016/j.ensm.2018.09.025

[S23] B. Jia, Q. Yu, Y. Zhao, M. Qin, W. Wang et al., Bamboo-like hollow tubes with MoS2/N-doped-C interfaces boost potassium-ion storage. Adv. Funct. Mater. **28**(40), 1803409 (2018). https://doi.org/10.1002/adfm.201803409

[S24] F. Yang, H. Gao, J. Hao, S. Zhang, P. Li et al., Yolk-shell structured FeP@C nanoboxes as advanced anode materials for rechargeable lithium-/potassium-ion batteries. Adv. Funct. Mater. **29**(16), 1808291 (2019). https://doi.org/10.1002/adfm.201808291

[S25] C. Yan, X. Gu, L. Zhang, Y. Wang, L. Yan et al., Highly dispersed Zn nanoparticles confined in a nanoporous carbon network: promising anode materials for sodium and potassium ion batteries. J. Mater. Chem. A **6**(36), 17371-17377 (2018). https://doi.org/10.1039/C8TA05297B

[S26] Y. Li, C. Yang, F. Zheng, X. Ou, Q. Pan et al., High pyridine N-doped porous carbon derived from metal–organic frameworks for boosting potassium-ion storage. J. Mater. Chem. A **6**(37), 17959-17966 (2018). https://doi.org/10.1039/C8TA06652C

[S27] H. Yamamoto, S. Muratsubaki, K. Kubota, M. Fukunishi, H. Watanabe et al., Synthesizing higher-capacity hard-carbons from cellulose for Na- and K-ion batteries. J. Mater. Chem. A **6**(35), 16844-16848 (2018). https://doi.org/10.1039/C8TA05203D

[S28] J. Bai, B. Xi, H. Mao, Y. Lin, X. Ma et al., One-step construction of N,P-codoped porous carbon sheets/cop hybrids with enhanced lithium and potassium storage. Adv. Mater. **30**(35), 1802310-1802318 (2018). https://doi.org/10.1002/adma.201802310

[S29] Q. Yu, B. Jiang, J. Hu, C.Y. Lao, Y. Gao et al., Metallic octahedral $CoSe_2$ threaded by n-doped carbon nanotubes: a flexible framework for high-performance potassium-ion batteries. Adv. Sci. **5**(10), 1800782 (2018). https://doi.org/10.1002/advs.201800782
